# Practical Design of 3,6-Di-*tert*-butyldiphenyldibenzofulvene Derivatives with Enhanced Aggregation-Induced Emission

**DOI:** 10.1021/acsaom.2c00067

**Published:** 2022-11-10

**Authors:** Carla Cunha, Mariana S. Peixoto, Joana Santos, Paulo E. Abreu, José A. Paixão, Marta Pineiro, J. Sérgio Seixas de Melo

**Affiliations:** † University of Coimbra, CQC-IMS, Department of Chemistry, Rua Larga, Coimbra 3004-535, Portugal; ‡ University of Coimbra, CFisUC, Department of Physics, Rua Larga, Coimbra 3004-516, Portugal

**Keywords:** 3,6-di-*tert*-butylfluorene, DPBF, aggregation-induced emission, time-resolved fluorescence, DLS, FLIM, X-ray crystal structure, molecular dynamics (MD) simulations

## Abstract

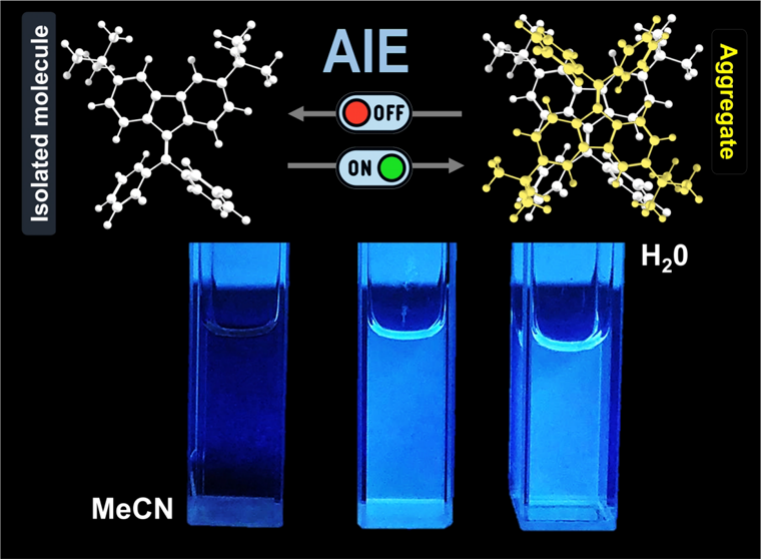

Diphenyldibenzofulvene derivatives consisting of an aromatic *tert*-butyl-substituted fluorene stator and different rotors consisting of nonsubstituted phenyl groups (**3,6-*dtb*-DPBF**) and monomethyl-substituted (**3,6-*dtb*-DPBFMe**) and dimethyl-substituted [**3,6-*dtb*-DPBF(Me)**_**2**_] forms have been synthesized and found to display aggregation-induced emission (AIE). The incremental number of substituents from **3,6-*dtb*-DPBF** to the **3,6-*dtb*-DPBFMe** and **3,6-*dtb*-DPBF(Me)**_**2**_ derivatives promotes significant changes, from a good solvent (acetonitrile, MeCN), where it is very poorly emissive, to thin films or aggregates, in MeCN/water mixtures, and a huge increment in fluorescence emission, which is found to be dependent on the water fraction, *f*_w_. The characteristics (size and distribution) of the aggregates were further corroborated with dynamic light scattering measurements. From time-resolved fluorescence experiments (TCSPC and FLIM), the increase in the contribution of the longer decay component is linked to the emission of the aggregate (AIE effect). To assist in the elucidation of the aggregation process at a molecular level, the data were complemented with computational studies [time-dependent density functional theory (TDDFT) and molecular dynamics (MD) simulations]. From MD, the octamer properly addresses the properties of the aggregate. As determined by the X-ray data, the crystal structure of a two-unit special disposition is identical to the geometry of the most stable structure obtained from MD and TDDFT calculations.

## Introduction

The enhancement of emission in the solid state has become a highly interesting field in the past several decades. In most of the emissive aromatic type systems, aggregation introduces π–π interactions that quench solid-state emissions, leading to the prevalent phenomenon (in the solid state) of aggregation-caused quenching (ACQ). In contrast with this phenomenon, aggregation-induced emission (AIE) is a phenomenon coined by Tang and co-workers in 2001 that reflects the large increase in the luminescence efficiency in solution of a bad solvent (aggregate), in comparison with a good solvent (dissolved monomer emission).^1,2^ Whereas in its early days the molecules displaying this effect (AIEgens) included the core structures hexaphenylsilole (HPS), tetraphenylethylene (TPE), and phenothiazine (PTZ), today it has matured and has expanded to many other families of molecules and polymers and, more recently, to diphenyldibenzofulvene derivatives (DPBFs).^3−5^

The interest in AIE research has led to a rapid growth of the structure and application of these molecules, as chemosensors, bioprobes, efficient organic light-emitting diodes, bioimaging agents, and solid-state emitters.^6−9^ Particular attention has been devoted to the rational design of these structures that involves a priori the correct knowledge of the mechanism behind this effect.^10−12^

Many reported AIEgens are designed to contain structurally hindered π-conjugated backbones, so that upon aggregation their fluorescence can be enhanced due to restricted intramolecular motion (RIM).^13,14^ However, rigidification, in contrast to the possibility of free rotation of the structure, may, in some systems, not be the sole requirement for emission to be seen in the solid or aggregate state. This has been the motive in some studies, in which a luminescence increment is observed and not necessarily solely due to intermolecular interactions, because the more generic term solid-state luminescence enhancement may apply.

**Scheme 1:**
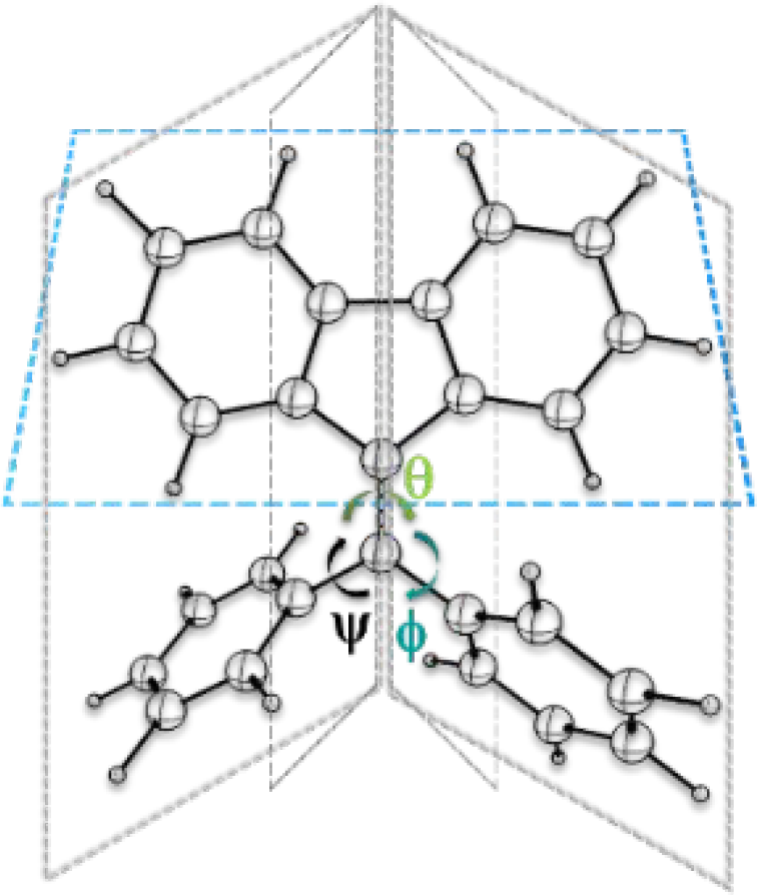
DPBF Structure with the Two Phenyl Rotors and the Fluorene Stator in a Three-Dimensional Perspective in Which θ, ψ, and ϕ Represent Internal Rotations That Can Be Used to Characterize Conformational Changes in DPBF θ corresponds to the rotation of the ethylenic C=C π-bond, and ψ and ϕ correspond to the rotations of the phenyl groups around the C–C σ-bonds.

DPBF derivatives (see Scheme 1), with two rotatable phenyl rings, constitute an intriguing class of AIEgens, featuring relatively simple molecular structures and straightforward synthesis, some of which show very interesting polymorphism-dependent emission, strong crystallization-induced emission enhancement (CIEE) effects, and thermochromic and mechanochromic fluorescence properties.^13,15^

Similar to silole derivatives (in the genesis of the AIE effect), these molecules are propeller-shaped, consisting of multiple phenyl rotors and olefinic or aromatic stators.^7^ DPBF is weakly fluorescent in a dilute acetonitrile solution, but in a mixture of 99% water, it forms aggregates leading to a 35-fold increase in the fluorescence quantum yield.^6−8^

In the structure of a dimer, as determined by crystal data, the dibenzofulvene stator and the phenyl rotors of DPBF are in an antiparallel orientation, with little involvement of π–π stacking interactions. Recently, we showed that the *tert*-butyl group strongly influences the AIE properties of the *tert*-butyl-TPE monomer and polymer structures.^16^ In this work, we use previously acquired knowledge to synthesize and investigate three DPBF derivatives with *tert*-butyl substituents in the fluorene core stator and an incremental number of methyl (in the diphenyl moiety rotor) groups. We found that such a wide range of AIEgens from the same fluorophore have rarely been reported. Interestingly, the *tert*-butyl substitute strongly influences the emissive properties, i.e., both the fluorescence quantum yields and lifetimes.

## Experimental Section

### Materials and Instrumentation

Chemicals were purchased from Sigma-Aldrich, ABCR GmbH, or TCI chemicals and used without further purification, unless described otherwise. For the photophysical studies and dynamic light scattering measurements, the solutions were prepared with solvents of spectroscopic grade or their equivalent: dry acetonitrile (MeCN, Uvasol, Merck), analytical grade chloroform (Fischer Chemical), or deionized water (18.2 MΩ cm at 25 °C, Milli-Q, Millipore). Deoxygenation was performed by bubbling the solutions with a stream of argon or nitrogen for approximately 20 min in a device described previously.^27^ Saturation with oxygen was achieved by bubbling the solutions with a stream of oxygen for 30 min. All measured solutions were freshly prepared (within the day) unless noted otherwise.

### Structural Characterization

Nuclear magnetic resonance (NMR) spectra were recorded at room temperature in deuterated chloroform (CDCl_3_) solutions on a Bruker Avance III instrument operating at 400.13 MHz for ^1^H and 100.61 MHz for ^13^C. Chemical shifts for ^1^H and ^13^C are expressed in parts per million relativel to an internal standard of tetramethylsilane. Coupling constants (*J*) are reported in hertz. High-resolution mass spectra (HRMS) were recorded on a Bruker microTOF-Focus mass spectrometer equipped with an electrospray ionization time-of-flight source. Melting points (mp) were determined in open glass capillaries. Thin-layer chromatography (TLC) analyses were performed using precoated silica gel.

### Photophysical Measurements

*Steady-State and Time-Resolved Photoluminescence*. For the photophysical experiments, all solvents used were of spectroscopic or equivalent grade. Absorption spectra were recorded in a 10 mm quartz cuvette in acetonitrile and an acetonitrile/water (% v/v) mixture on a Shimadzu UV-2600 spectrophotometer or an Agilent Cary 5000 UV–vis–NIR spectrometer (solid-state thin films). Fluorescence spectroscopic studies were performed using a Horiba-Jobin-Yvon Fluorolog 3.22 instrument. Fluorescence spectra were corrected for the wavelength response of the system with the appropriate correction files obtained for the instrument.

The fluorescence quantum yields (ϕ_F_) of all compounds, in solution and in thin films, were measured using the absolute method with a Hamamatsu Quantaurus QY absolute photoluminescence quantum yield spectrometer (model C11347, integration sphere). A clean sapphire substrate was used as a reference for the ϕ_F_ measurements of solid-state thin films. The color parameters were determined according to the CIE (Commission Internationale de l’Eclairage proceedings) 1931 scale diagram.^28^ The *x* and *y* color parameters were determined with the parameters provided by Quantaurus QY software obtained with the integration of the PL emission spectrum of each sample.

Fluorescence decays were measured using a home-built picosecond time-correlated single-photon counting (ps-TCSPC) apparatus described previously.^29^ The excitation source consisted of a tunable picosecond Spectra-Physics mode-lock Tsunami laser (Ti:sapphire, model 3950, 80 MHz repetition rate, tuning range of 700–1000 nm), pumped by a 532 nm continuous wave Spectra-Physics Millennia Pro-10s laser. The excitation wavelength (λ_exc_ = 261 nm) was obtained with a Spectra-Physics harmonic generator (model GWU-23PS). To eliminate the quantity of dispersed light, an RG530 filter was used after the sample holder and before the emission monochromator. Temperature control was achieved using a home-built system based on cooled nitrogen and electric heating. The fluorescence decay curves were deconvoluted using the experimental instrument response function signal collected with a scattering solution (aqueous Ludox solution). The deconvolution procedure was performed using the modulation function method, as implemented by G. Striker in the SAND program, and previously reported in the literature.^30^

#### FLIM (fluorescence lifetime imaging microscopy)

Fluorescence lifetime images were recorded by using a Becker and Hickl (GmbH) DCS-120 Confocal FLIM System. The system is equipped with a TCSPC-System module (SPC-150N) and a NIKON Ti2-U inverted optical microscope, controlled by a galvo-drive unit (Becker and Hickl GDA-121). Three objectives are available (20×, 40×, and 100×). The DCS-121 confocal microscope system was equipped with a polarizing beam splitter. Also, a hybrid GaAsP photodetector (300–720 nm), controlled by a DCC-100 detector controller card, was used as a detector. The excitation source is a picosecond diode laser with a wavelength of 375 nm (bh BDL series lasers) working on a pulsed mode (repetition rate of 80 MHz). The IRF of the system is found to be <100 ps. The total laser power at the sample was set to 40% of the maximum value, and the collected emission passed through a 1 mm pinhole, a long-pass filter (ET390), and a band-pass filter (ET550/60). The FLIM images were scanned and recorded at a resolution of 512 × 512 pixels using the “FIFO imaging” mode of the SPC-150N modules. Data were analyzed with SPCImage NG data analysis software. The decays were fitted using the maximum-likelihood algorithm [or maximum-likelihood estimation (MLE)] fitting method in the individual pixels. The sapphire substrates were glued on a microscope slide, and the measurements were performed by placing the inverted slide on the microscope stage.

#### Dynamic Light Scattering (DLS) Measurements

Dynamic light scattering studies were performed using a Zetasizer Nano ZS (Malvem Panalytical). The size distribution of the aggregates was measured in 10 mm quartz cuvettes with a final volume of 1 mL, at 20 °C, in three consecutive runs of the same sample, using freshly prepared solutions. The refractive index and viscosity of the acetonitrile/water mixtures were determined in advance at the experimental temperature and were seen to be in agreement with those found in the literature for different reported temperatures.^31^

#### Solutions and Film Preparation

An appropriate amount of powder of each compound was diluted in recently dried acetonitrile (MeCN) with absorption of 0.5–0.6 in a 10 mm quartz cuvette. Then, 100 μL of the stock solution was diluted with the proper amount of MeCN or the MeCN/water mixture to obtain the desired water fraction [*f*_w_ = 0–95% (v/v)] in a final volume of 2 mL. The photophysical studies of the resultant mixtures were performed immediately after sample preparation. Thin films from the compounds were obtained with a desktop precision spin-coating system (model P6700 series from Speedline Technologies), as described previously.^31^ Briefly, thin films from the samples were obtained by deposition of ∼50 μL from a solution of the compounds into a circular sapphire substrate (12 mm diameter) followed by spin-coating (2500 rpm for 60 s) in a nitrogen-saturated atmosphere (2 psi). The solutions for spin-coating were prepared by adding 2 mg of the samples along with 15 mg of Zeonex to 200 μL of a chloroform solution, with stirring, at the environmental temperature, overnight.

### X-ray Diffraction

Single crystals suitable for X-ray diffraction analysis were grown for **3,6-*dtb*-DPBF** by slow diffusion in an ethanol solution of the compound. The crystal data and experimental details for data collection are given below. Single-crystal X-ray data for **3,6-*dtb*-DPBF** were collected at room temperature on a small crystal that turned out to be a two-component twin using graphite monochromated Mo Kα (λ = 0.71073 Å) radiation on a Bruker APEX II diffractometer. Structure determination and refinement were performed with SHELXT-2018–2/SHELXL-2018-3; structure validation was performed with PLATON, and ORTEP illustrations and drawings of packing diagrams were created with Mercury. The crystallographic details are listed in Table S1. CIF files containing the supplementary crystallographic data were deposited at the Cambridge Crystallographic Data Centre, with reference 2193638.

### Time-Dependent Density Functional Theory (TDDFT) Studies

All theoretical calculations were of the DFT type, carried out using GAMESS-US^32^ version R3. In the TDDFT calculation of FC (Franck–Condon) excitations, the dielectric constant of the solvent was split into a “bulk” component and a fast component, which is essentially the square of the refractive index. Under “adiabatic” conditions, only the static dielectric constant is used. A 6-31G** basis set was used in either DFT or TDDFT calculations.

### Molecular Dynamics (MD) Simulations

MD simulations were performed to assist in the interpretation of the photophysical experimental results for the aggregation of **3,6-*dtb*-DPBF**, **3,6-*dtb*-DPBFMe**, and **3,6-*dtb*-DPBF(Me)**_**2**_ in various media: water, MeCN, and MeCN/water mixtures [25:75, 40:60 (v/v)] (see Table S4). For the simulations of the **3,6-*dtb*-DPBF** molecule, we considered solute concentrations of 0.0025, 0.0050, 0.0075, 0.0100 mol dm^–3^, corresponding to a total of two, four, six, and eight solute molecules, respectively. Afterward, we extended the investigation to **3,6-*dtb*-DPBFMe** and **3,6-*dtb*-DPBF(Me)**_**2**_, and the solute concentration was kept at 0.0100 mol dm^–3^ (eight solute molecules) (see Table S3).

All equilibrium MD simulations were carried out using the GROMACS-2019 package.^33,34^ To establish the interactions involving the solute molecules, we used the AMBER force field with GAFF2 parameters,^35^ and the topology of these molecules was constructed following the standard protocol for the AMBER force field. The structures were optimized at the DFT-B3LYP/6-31G* level of theory with GAMESS.^36^ The partial charges, used in the description of the electrostatic interactions, were derived according to the established RESP fitting procedure, as implemented in RED^36^ [see Figure S19 for the three-dimensional (3D) structures of the solute molecules and Tables S5–S7 for the corresponding atomic charges]. Acpype^37,38^ was used to obtain the geometry and topology input files. To prepare the system for simulation, the solute molecules were placed, separated, in the center of a cubic box whose dimensions were adjusted to achieve the required solution concentration. Finally, the solute molecules were solvated with water, MeCN, or a MeCN/water mixture (25:75, 40:60). The water molecules were described using the TIP4P-2005 model,^39^ and the acetonitrile was described using the parametrization described in ref (40). This was followed by an initial energy minimization of the entire system in the simulation box to reduce undesirable repulsions. Afterward, to relax the system to the appropriate temperature and pressure, in the equilibrium phase, two consecutive short simulations of 1 ns were performed using the *NVT* and *NPT* ensembles, respectively. The temperature was set to 298.15 K using the velocity-scaling thermostat^41^ (coupling time of 0.1 ps), and the pressure set to 1 bar by employing the Parrinello–Rahman barostat^42^ (coupling time of 2 ps).

The production step of the MD simulations was carried out by running one trajectory of each system under the *NVT* ensemble, for 500 ns (**3,6-*dtb*-DPBFMe**) or 200 ns [**3,6-*dtb*-DPBFMe** and **3,6-*****dtb*****-DPBF(Me)**_**2**_]. For all dynamic calculations, the Leapfrog algorithm with a time step of 2 fs was used to integrate the equations of motion, while the Linear Constraint Solver (LINCS)^43^ scheme was used to impose bond constraints. The particle mesh Ewald method^44,45^ was employed to estimate the long-range electrostatic energy; a cuttoff of 10 Å was applied for both Coulomb and van der Waals interactions, and periodic boundary conditions were employed in all simulations.

To characterize the aggregate and its mechanism of formation, we calculated some relevant properties, such as the distances between molecules, radial distribution functions (rdf), cluster analysis, and cluster size, with the software available in GROMACS-2019^33,34^ and visual inspection was performed using VMD.^26^ For the calculation of the aggregate/cluster size throughout the trajectory, the time, and the relative frequency of each cluster, molecules were arbitrarily considered to be clustered if the minimum distance between molecules (the distance of closest approach) was <3.5 Å. For the estimation of the most probable structure of each aggregate of eight elements during the MD simulation, we employed cluster analysis for the section of trajectory where the cluster was fully formed, with a root-mean-square deviation cutoff of 3 Å.

### Synthesis and Structural Characterization of the 3,6-*dtb*-Diphenyldibenzofulvene Derivatives

3,6-*dtb*-Diphenyldibenzofulvene derivatives (3,6-*dtb*-DPBFs) were synthesized through the condensation of 3,6-*dtb*-fluorenone and the appropriate benzophenone following the previously described methodology.^3^ To a 50 mL round-bottom flask with 10 mL of dry THF were added 3,6-di-*tert*-butlyfluorene (1.5 mmol) and NaH (7.5 mmol). The reaction mixture was stirred under reflux for 4 h at 80 °C. Then, 2.5 mmol of the selected benzophenone was added, and the reaction mixture was left to reflux for 48 h. After cooling to room temperature, the mixture was purified by liquid–liquid extraction with ethyl acetate and water (three times). The collected organic fractions were dried with Na_2_SO_4_ anhydride, and the organic solvent was evaporated under reduced pressure. The desired compounds were obtained after recrystallization from dry THF and ethanol at −8 °C. Details of the synthesis of each 3,6-*dtb*-DPBF derivative and characterization by ^1^H and ^13^C NMR and high-resolution mass spectrometry HRMS(ESI) are presented in the Supporting Information (see Figures S1–S9).

#### Synthesis of 3,6-Di-*tert*-butyldiphenyldibenzofulvene (3,6-*dtb*-DPBF)

First, 2.5 mmol of benzophenone was added to the reaction mixture after it had been heated at 80 °C for 4 h. A pale-yellow solid was obtained in 10.5% yield: ^1^H NMR (400 MHz, CDCl_3_) δ 7.70 (d, *J* = 1.7 Hz, 2H), 7.37 (m, 10H), 6.96 (dd, *J* = 8.4, 1.9 Hz, 2H), 6.52 (d, *J* = 8.3 Hz, 2H), 1.35 (s, 18H); ^13^C NMR (101 MHz, CDCl_3_) δ 150.9, 143.9, 143.2, 140.7, 136.6, 133.3, 129.7, 128.7, 127.9, 124.4, 123.7, 115.8, 34.9, 31.5; HRMS (ESI) *m*/*z* 443.2705 (found), 443.2733 (calculated for C_34_H_34_ [M + H]^+^); mp >300 °C.

#### Synthesis of 3,6-Di-*tert*-butyldiphenyldibenzofulvene (**3,6-*dtb*-DPBFMe**)

First, 2.5 mmol of 4-methylbenzophenone was added to the reaction mixture after it had been heated at 80 °C for 4 h. A pale-yellow solid was obtained in 2.1% yield: ^1^H NMR (400 MHz, CDCl_3_) δ 7.70 (d, *J* = 8 Hz, 2H), 7.38 (m, 5H), 7.20 (d, *J* = 8.0 Hz, 4H), 6.96 (m, 2H), 6.64 (d, *J* = 8.3 Hz, 1H), 6.50 (d, *J* = 8.4 Hz, 1H), 2.42 (s, 3H), 1.35 (s, 18H); ^13^C NMR (101 MHz, CDCl_3_) δ 150.8, 143.4, 140.6, 140.2, 137.8, 136.7, 129.7, 129.4, 128.7, 127.7, 123.6, 115.8, 34.9, 31.5, 21.4; HRMS (ESI) *m*/*z* 457.2859 (found), 457.2890 (calculated for C_35_H_36_ [M + H]^+^); mp >300 °C.

#### Synthesis of 3,6-Di-*tert*-butyldiphenyldibenzofulvene [**3,6-*dtb*-DPBF(Me)_2_**]

First, 2.5 mmol of 4,4-dimethylbenzophenone was added to the reaction mixture after it had been heated at 80 °C for 4 h. A pale-yellow solid was obtained in 3.4% yield: ^1^H NMR (400 MHz, CDCl_3_) δ 7.70 (d, *J* = 1.7 Hz, 2H), 7.21 (m, 8H), 6.98 (dd, *J* = 8.4, 1.9 Hz, 2H), 6.62 (d, *J* = 8.4, 2H), 2.41 (s, 6H), 1.35 (s, 18H); ^13^C NMR (101 MHz, CDCl_3_) δ 150.6, 143.9, 140.6, 140.5, 137.7, 136.8, 133.3, 129.8, 129.4, 124.3, 123.6, 34.9, 31.5, 21.4; HRMS (ESI) *m*/*z* 471.3017 (found), 470.2974 (calculated for C_36_H_38_ [M + H]^+^); mp >300 °C.

## Results and Discussion

### Synthesis

**Scheme 2:**
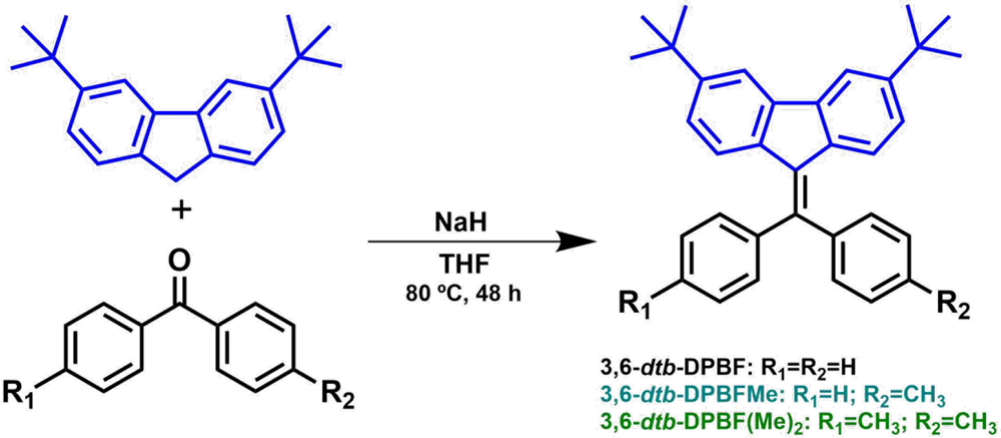
General Synthetic Route, Structures, and Acronyms of 3,6-*dtb*-Diphenyldibenzofulvene Derivatives [**3,6-*dtb*-DPBF**, **3,6-*dtb*-DPBFMe**, and **3,6-*dtb*-DPBF(Me)_2_**]

Spectroscopically pure 3,6-*dtb*-diphenyldibenzofulvene derivatives were obtained after recrystallization in a THF/EtOH solvent, using a one-pot chromatography-free procedure for the condensation of 3,6-di-*tert*-butylfluorene and benzophenone (see Scheme 2). See the Supporting Information for the a complete description of the synthesis, structural characterization, and further experimental details (Figures S1–S9).

**Figure 1:**
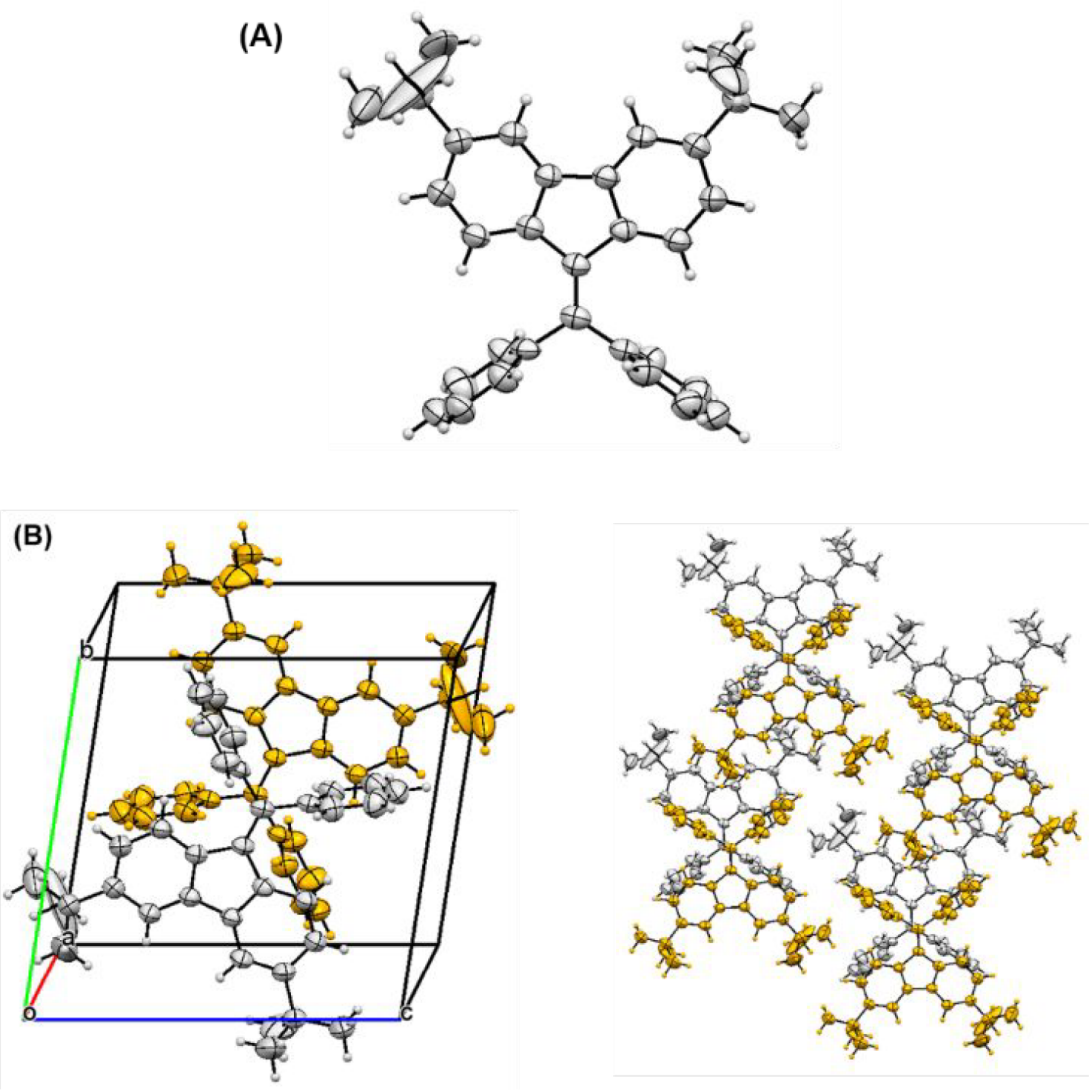
(A) Molecular structure of compound **3,6-*dtb*-DPBF** (asymmetric unit) depicting the anisotropic displacement ellipsoids at the 50% probability level. (B) Unit cell (left) and molecular packing (right) of **3,6-*dtb*-DPBF**.

Crystals for X-ray analysis were grown for **3,6-*dtb*-DPBF** by slow evaporation from an ethanol solution. The crystal structure was determined by single-crystal X-ray diffraction using a small needle crystal that turned out to be a two-component twin. The **3,6-*dtb*-DPBF** compound crystallizes in triclinic space group *P*1̅, with a single molecule in the asymmetric unit (Figure 1A and Table S1 for further crystallographic details). The molecule has approximate *C*_2*v*_ symmetry, the two phenyl rings being almost perpendicular to the dibenzofulvene core [88.39(17)° and 84.18(17)° for rings C23>C28 and C29>C34, respectively], the angle between the two phenyl rings being 72.54(16)°. The large displacement ellipsoids of the terminal C atoms of the *tert*-butyl group indicate a significant degree of disorder of these groups, probably arising from large thermal librations around the C–C aryl bond, which is not uncommon for such a group. The unit cell, shown in Figure 1B, contains two molecules related by an inversion center. Inspection of the 3D packing shows intermolecular short contacts involving interactions of the C···H···π type with H–Cg (Cg = ring centroid) distances in the range of 2.79–2.98 Å, as depicted in Figure S10.

The crystal structure is free of solvent molecules (the solvent accessible volume in the unit cell is only 68 Å).

### Photophysical Studies

**Figure 2:**
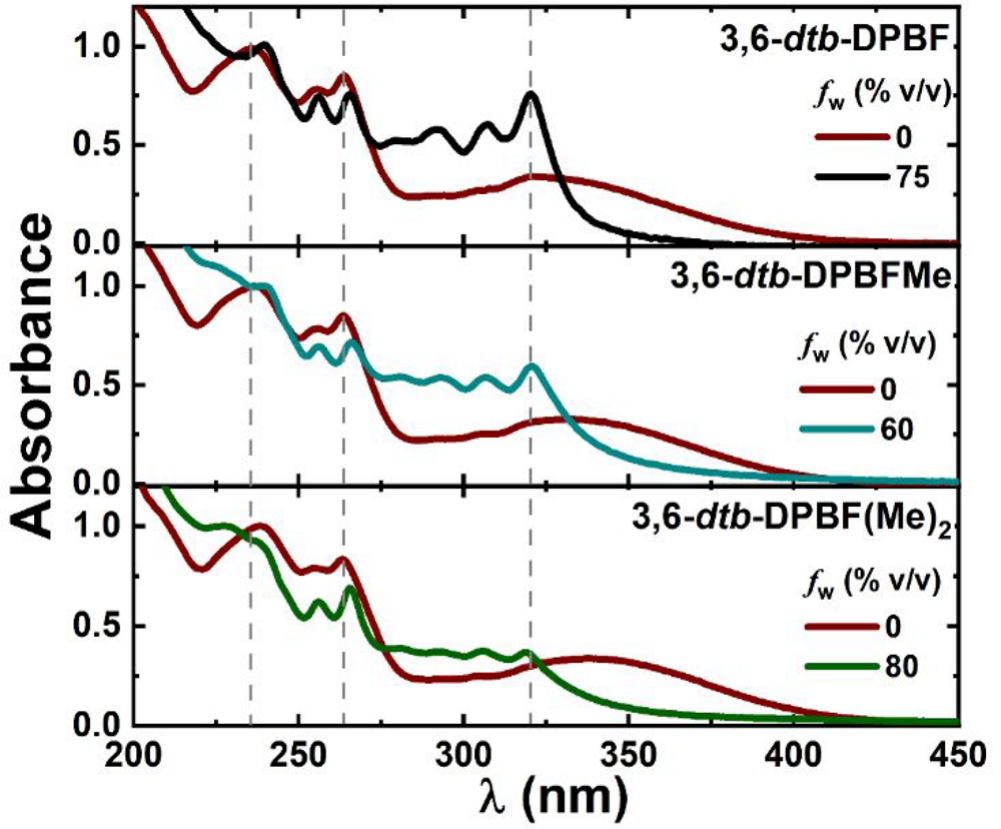
Dependence of the absorption spectra of 3,6-*dtb*-DPBF derivatives on the water content in MeCN/water mixtures (in MeCN and aggregate solution). *f*_w_ = 75% for **3,6-*dtb*-DPBF**. *f*_w_ = 60% for **3,6-*dtb*-DPBFMe**. *f*_w_ = 80% for **3,6-*dtb*-DPBF(Me)**_**2**_. *f*_w_ is the volume percentage of water in the mixtures.

**Figure 3:**
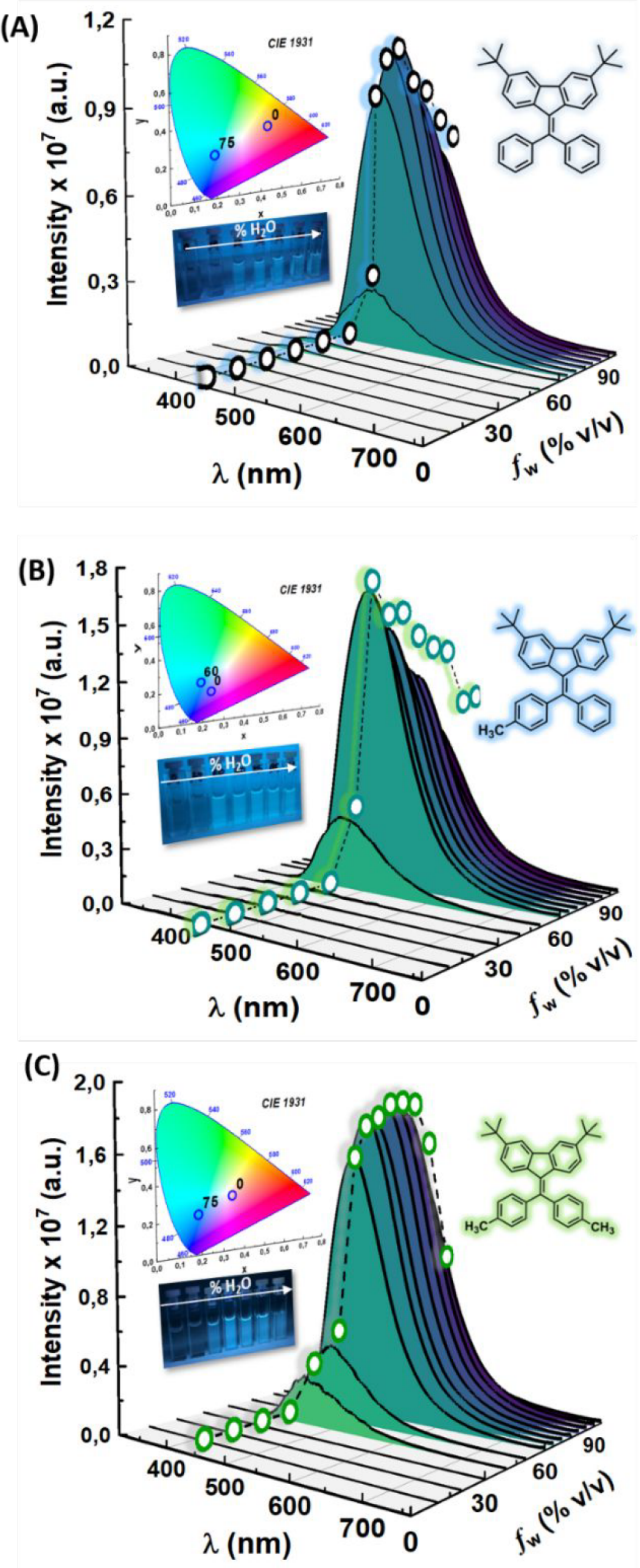
Room-temperature fluorescence emission spectra for the 3,6-*dtb*-DPBF derivatives (A) **3,6-*dtb*-DPBF**, (B) **3,6-*dtb*-DPBFMe**, and (C) **3,6-*dtb*-DPBF(Me)**_**2**_ in MeCN/water mixtures (λ_exc_ = 320 nm) and the respective correlations of emission area with an increasing water fraction (*f*_w_ = 0–95%). Photoluminescence color coordinates of the 3,6-*dtb*-DPBF derivatives in acetonitrile and when aggregated in solution (MeCN/water mixtures) plotted in the CIE 1931 chromaticity diagram and photos under ultraviolet irradiation (with λ_exc_ = 254 nm) of the fluorescence emission of the 3,6-*dtb*-DPBF derivatives in MeCN/water mixtures [from left (0%) to right (95%) with increments of water fraction (% v/v)].

The photophysical properties and the presence of aggregates leading to AIE were further investigated with the 3,6-*dtb*-DPBF derivatives. In Figure 2, the absorption spectra of the compounds in acetonitrile and MeCN/water mixtures, for the water fraction in which the compound has its highest fluorescence quantum yield (ϕ_F_), clearly show the difference indicative of the formation of aggregates in the water content mixtures (Figure 3). As found for other DPBF derivatives,^3,17,18^ the increase in *f*_w_, and consequently the change from a good to a poor solvent medium, leads to a change in 3,6-*dtb*-DPBF from an isolated molecule to an emissive aggregate. Data in Figure 3 (summarized in Table 1) show that the compounds are poorly emissive in acetonitrile, becoming emissive when *f*_w_ ≥ 60%, with maximum fluorescence emission efficiency (ϕ_F_ in Table 1) at *f*_w_ values of 75% (**3,6-*dtb*-DPBF**), 60% (**3,6-*dtb*-DPBFMe**), and 80% for [**3,6-*dtb*-DPBF(Me)**_**2**_] (Figure 3). Indeed, in acetonitrile, rotational freedom of the two phenyl rotors is present in the 3,6-*dtb*-DPBF molecules (in the excited state, the bond connecting these rotors to the fluorene stator gains single-bond character), leading to the dominance of the radiationless deactivation channel, whereas in poor solvents (MeCN/water mixtures for which *f*_w_ > 60%), the aggregate prevents rotation of the rotors, thus leading to the dominance of radiative deactivation.

**Table 1:** Room-Temperature Spectroscopic Data (absorption and fluorescence emission maxima together with Stokes shifts, Δ_SS_) Along with Fluorescence Quantum Yields (ϕ_F_) for the 3,6-*dtb*-DPBF Derivatives in Acetonitrile (MeCN) and Aggregated (agg) in Solution^a^

compound	medium	λ_abs_ (nm)	λ_em_ (nm)	Δ_SS_ (cm^–1^)	ϕ_F_
**DPBF**^b^	MeCN	234	420	6772	4 × 10^–5^
		258			
		327			
	agg (*f*_w_ = 70%)	237	470	10672	0.110
		267			
		313			
**3,6-*dtb*-DPBF**	MeCN	240	ND	ND	ND
		264			
		322			
	agg (*f*_w_ = 75%)	240	465	9647	0.120
		266			
		321			
**3,6-*dtb*-DPBFMe**	MeCN	237	ND	ND	ND
		264			
		331			
	agg (*f*_w_ = 60%)	239	468	9785	0.171
		266			
		321			
**3,6-*dtb*-DPBF(Me)**_**2**_	MeCN	238	ND	ND	ND
		263			
		338			
	agg (*f*_w_ = 80%)	228	463	9750	0.216
		264			
		319			

a Values obtained for previously studied diphenyldibenzofulvene (DPBF) are included for comparison. ND, fluorescence not detected, meaning that, within our instrumental facilities, the ϕ_F_ is <10^–5^.^19^

b Data from ref (3).

The complete set of electronic spectral data (including wavelength maxima for absorption, fluorescence emission, fluorescence quantum yields, ϕ_F_, and Stokes shifts, Δ_SS_) is summarized in Table 1. Literature data for the nonsubstituted diphenyldibenzofulvene (DPBF) is also included for comparison.^3^

Figure 3 shows that the emission spectra of the different 3,6-*dtb*-DPBF aggregates (observed for *f*_w_ > 60%) show emission maxima in the range of ∼463–468 nm (see Table 1). The incremental increase in the amount of water leads to a gradual increase in the total fluorescence quantum yield (ϕ_F_) of the three 3,6-*dtb*-DPBF derivatives to a water fraction of 60% (see Figure S11). The ϕ_F_ values are relatively high for the aggregate in solution (12–22%), with values of ∼15% in thin films (see Table 1). Methyl groups, in the phenyl rotor, are shown to influence the emission intensity, both when aggregated in solution and in thin films, leading to an increase in the fluorescence quantum yield. Indeed, **3,6-*dtb*-DPBF(Me)**_**2**_ shows the highest ϕ_F_ value of the three derivatives, reaching 22% of fluorescence emission when *f*_w_ = 80%. Additionally, the CIE coordinates for the emission spectra were obtained and found to be quite identical for the three compounds (aggregates in solution) (see Figure 3B and Table S2).

### Dependence with *f*_w_, from Time-Resolved Fluorescence

To further characterize and rationalize the deactivation pathways of the first singlet excited state, time-resolved fluorescence studies were performed in acetonitrile/water mixtures. The photoluminescence (PL) intensity response with time, *I*(*t*), is given by eq 1, with decay times, τ_*i*_, and pre-exponential factors, *a_i_*, where *i* stands for the number of the exponential
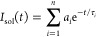
1where τ_*i*_ terms are the decay times and *a_i_* terms are the pre-exponential factors that represent the contribution of each exponential term to *t* = 0. The fractional contribution of each species [*C_i_* (%)] was determined using eq 2:^20^
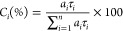
2where *n* represents the number of exponential terms, *a*_*i*_ the contribution of each exponential term, for *t* = 0, and τ*_i_* the associated decay time.

**Figure 4:**
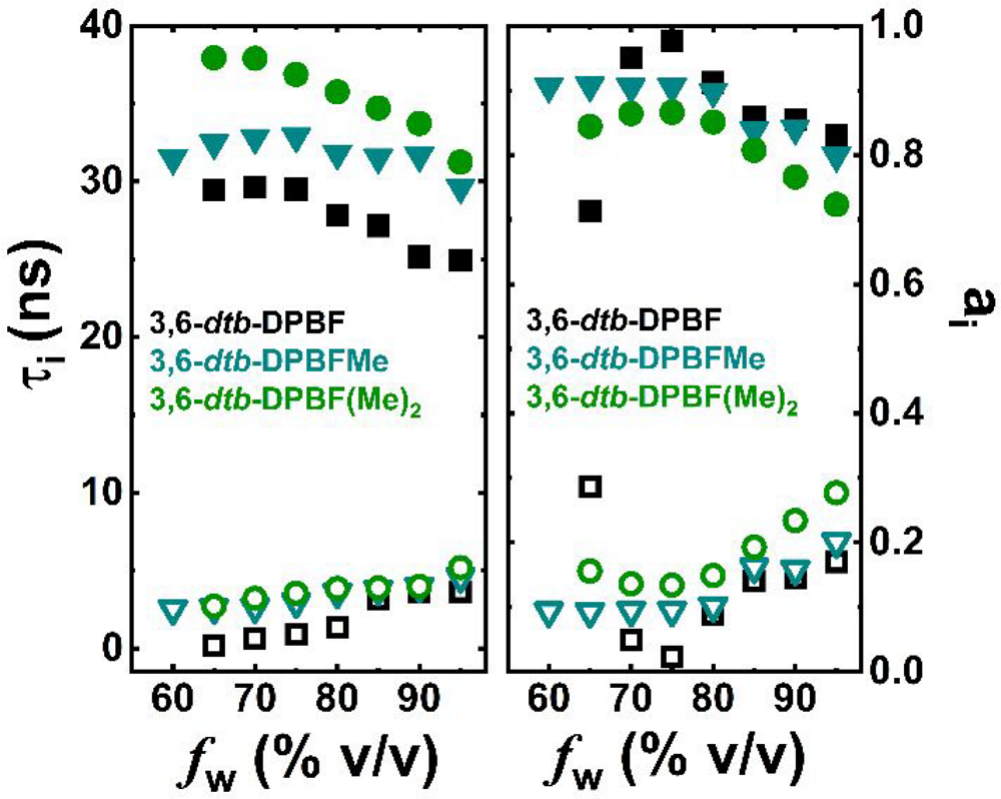
Fluorescence decays times (τ_*i*_) and pre-exponential factors (*a_i_*) for the 3,6-*dtb*-DPBF derivatives obtained with the ps-TCSPC technique in MeCN/water mixtures, with different water fractions, *f*_w_, at 293 K, with λ_exc_ = 261 nm and λ_em_ = 470 nm. Legend: empty symbols, monomer lifetimes; filled symbols, aggregate lifetimes.

The obtained data and analysis in Figure 4 and Figures S12 and S13 show that the fluorescence decays are, in MeCN/water mixtures, well-fitted with double-exponential decay laws with decay time values of τ_1_ and τ_2_. With τ_1_ increasing and τ_2_ decreasing with *f*_w_, an increase in *f*_w_ (with *f*_w_ > 65% in water content) leads to a decrease in the pre-exponential factor (*a*_1_) associated with the shorter decay time (τ_1_) with a concomitant increase in the pre-exponential factor (*a*_2_) associated with the longer component (τ_2_) (see Figure 4). The *a*_2_, associated with the longer decay time (τ_2_) and with a larger contribution (% *C*_2_) at higher *f*_w_ values, is therefore associated with aggregate emission. More interesting is the observation of the dependence of the contribution of the τ_2_ decay time component (*C*_2_) with an increase in *f*_w_ (Figure S13). Indeed, the % *C*_2_ follows the trend observed with ϕ_F_.

From Figure 4, we can conclude that the monomer lifetimes (τ_1_) vary from 0.15 to 2.94 ns whereas those associated with aggregates (τ_2_) vary from ∼30 to 38 ns. The methyl groups, at the phenyl rotor, have little influence on the lifetime values of the monomer component. However, the lifetime of the aggregates increases with the introduction of the methyl substituents, thus showing that this can be used to model the fluorescence properties of DPBF derivatives. This can also be correlated with the DLS experiments (next section) demonstrating the presence of a higher number of fluorescent aggregates at higher *f*_w_ values.

### Dynamic Light Scattering

**Figure 5:**
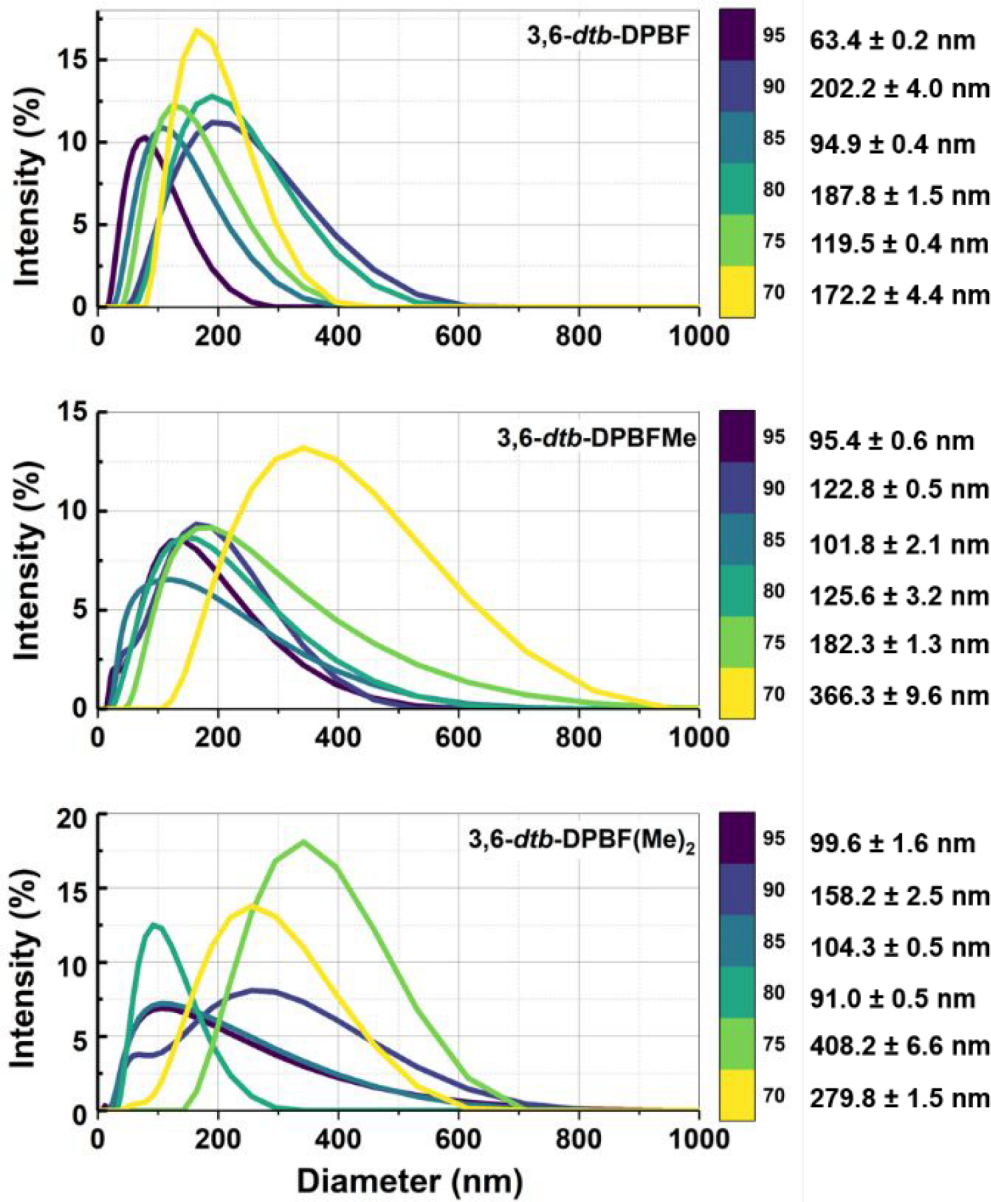
DLS particle size distribution curves obtained in MeCN/water (>70–95% H_2_O, % v/v) mixtures for the 3,6-*dtb*-DPBF derivatives. The count rates (% intensity) and the mean values of the hydrodynamic radius with MeCN/water mixtures, with different water fraction values, *f*_w_, are displayed.

The results of dynamic light scattering (DLS) experiments conducted for all of the 3,6-*dtb*-DPBF derivatives at different *f*_w_ values are summarized in Figure 5 and Figure S14 and were obtained only when aggregates, and therefore AIE, could be observed. For *f*_w_ < 70%, a poor autocorrelation (polydispersity is very high) is observed due to low scattering intensity (see Figure S14) and therefore poor aggregate formation. For *f*_w_ > 70–95%, it is possible to determine the hydrodynamic radius (Figure 5) and the autocorrelation function (observing a good signal-to-noise ratio) is obtained (Figure S14). The count rate and the mean hydrodynamic radius (*R*_h_)^21^ vary depending on *f*_w_, indicative of aggregates of different sizes. These aggregates displayed average diameters of ∼63–202 nm (**3,6-*dtb*-DPBF**), 95–366 nm (**3,6-*dtb*-DPBFMe**), and 100–408 nm [**3,6-*dtb*-DPBF(Me)**_**2**_].

From the data presented above, one can see that (i) differences exist in the absorption and emission maxima in MeCN/water mixtures and in thin films between the monosubstituted (CH_3_) and disubstituted [(CH_3_)_2_] derivatives (between DPBF^3^ and **3,6-*dtb*-DPBF**), (ii) this is also mirrored in the photophysical parameters, including fluorescence quantum yields, and (iii) particle aggregate sizes determined by DLS constitute evidence that the 3,6-*dtb*-DPBF derivatives in their aggregate form (with different *f*_w_ values) critically depend on the presence of *tert*-butyl groups, in positions 3 and 6, which leads to the adoption of different structural orientations (conformations) of the aggregate.

### Dependence on O_2_ Saturation and N_2_ Saturation of the Time-Resolved Fluorescence Data

Additional time-resolved fluorescence experiments in MeCN/water mixtures were performed in N_2_-saturated (without dissolved oxygen) and oxygen-saturated (O_2_ sat.) solutions, and the results are presented in Table 2 and Figure S15. These were obtained at *f*_w_ values with the highest ϕ_F_ value. It is possible to observe that the emissive species, previously associated with aggregate emission, shows longer decay times with values of ∼27–37 ns. In these experiments, all solutions were prepared in a glovebox under a N_2_-saturated atmosphere. The insensitivity of the fluorescence decay time of the aggregate toward O_2_ seems to indicate that the “emissive unit” is protected from the encounter with the molecular oxygen dissolved in the solvent [[O_2_] in MeCN of 9.1 mmol L^–1^ and [O_2_] in H_2_O of 1.39 mmol L^–1^ (at 20 °C)].^22,23^ However, the fact that the fluorescence quantum yield strongly decreases (quenching of fluorescence) in the presence of concentrated oxygen (in O_2_-saturated solutions) indicates that the “emissive unit” should be considered to be constituted by a network of molecules that make (in the aggregate) different contributions to the total emission, making diffusional quenching by oxygen less effective. The emissive structures (in the presence of oxygen) keep the essence of its decay channel and therefore its τ_F_ value.

**Table 2:** Fluorescence Quantum Yields (ϕ_F_), Fluorescence Decay Times (τ_1_ and τ_2_), and Pre-exponential Factors (*a*_1_ and *a*_2_), for the 3,6-*dtb*-DPBF Derivatives in MeCN/Water Mixtures with Oxygen, without Oxygen (N_2_-saturated), and with Oxygen Saturation (O_2_ sat.) at 293 K, Obtained with λ_exc_ = 261 nm and λ_em_ = 470 nm

compound	conditions	ϕ_F_	τ_1_ (ns)	τ_2_ (ns)	*a*_1_ (% C_1_)	*a*_2_ (% C_2_)	χ^2^
**3,6-*dtb*-DPBF** (*f*_w_ = 75%)	N_2_ sat.	0.104	1.02	29.45	0.045 (0.2)	0.955 (99.8)	1.03
	air	0.120	0.90	29.49	0.023 (0.1)	0.977 (99.9)	1.11
	O_2_ sat.	0.053	1.45	27.39	0.231 (2)	0.769 (98)	1.02
**3,6-*dtb*-DPBFMe** (*f*_w_ = 60%)	N_2_ sat.	0.139	2.46	32.83	0.099 (1)	0.901 (99)	1.05
	air	0.171	2.55	33.02	0.101 (1)	0.899 (99)	1.05
	O_2_ sat.	0.116	2.55	32.81	0.150 (1)	0.850 (99)	1.03
**3,6-*dtb*-DPBF(Me)**_**2**_ (*f*_w_ = 80%)	N_2_ sat.	0.196	3.85	36.87	0.134 (2)	0.866 (98)	1.05
	air	0.216	3.78	37.16	0.146 (2)	0.854 (98)	1.09
	O_2_ sat.	0.117	2.96	33.48	0.264 (3)	0.736 (97)	0.99

With the methyl groups, in the phenyl rotor [**3,6-*dtb*-DPBFMe** and **3,6-*dtb*-DPBF(Me)**_**2**_], the two decay times increase with air dissolved in the solution and the increase of the oxygen concentration has a weaker impact than **3,6-*dtb*-DPBF** (the fluorescence quantum yield decreases 50%). **3,6-*dtb*-DPBFMe**, with one methyl group, shows that the decay time remains unchanged and the fluorescence quantum yield decreases 17%. **3,6-*dtb*-DPBF(Me)**_**2**_, with two methyl groups, shows that the fluorescence quantum yield decreases 40%, from N_2_- to O_2_-saturated solutions.

### Results of MD Simulations

Initially, to investigate the aggregation of **3,6-*dtb*-DPBF** in the various selected media, we performed MD simulations with two, four, six, and eight solute molecules, solvated by acetonitrile/water mixtures (25:75, 40:60 v/v), water, and acetonitrile (see Figure S16 for the MD results of these trajectories). The results show a full aggregation at all four concentrations in water and MeCN/water mixtures. In Figure S16 (top panel), it is possible to observe the total aggregation of all solute molecules used in the simulation, which took place at the beginning of each simulation (30–90 ns), except for the lower concentration, where it happens around 200 ns. These results validate the model as this mimics the behavior observed experimentally. Moreover, because the objective is to fully characterize the aggregation dynamics, eight was established as the suitable number of solute molecules for describing the aggregation process, thus ensuring a good balance between system representativeness and computational time and cost. Furthermore, due to the early formation of the octamer and the structural similarity of the **3,6-*dtb*-DPBFMe** and **3,6-*dtb*-DPBF(Me)**_**2**_ molecules to **3,6-*dtb*-DPBF**, 200 ns simulations were established to be sufficient for the simulations.

**Figure 6:**
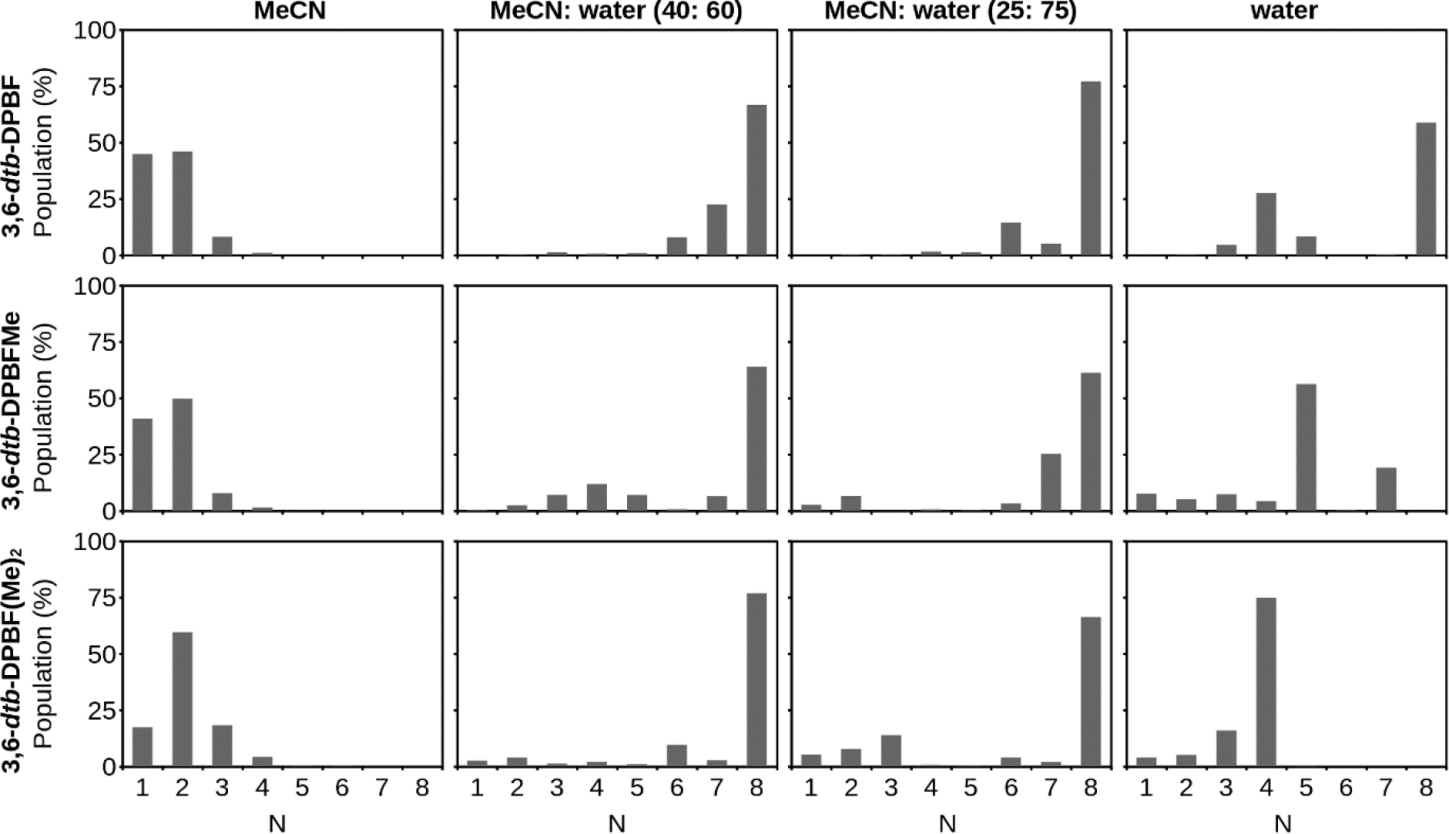
Normalized histograms of the cluster size of the **3,6-*dtb*-DPBF**, **3,6-*dtb*-DPBFMe**, and **3,6-*dtb*-DPBF(Me)**_**2**_ simulations showing the relative populations of molecules in aggregates of different sizes. All four solvents were considered in this analysis: acetonitrile (left), 40:60 MeCN/water (second), 25:75 MeCN/water (third), and water (right). The plots are in order of increasing polarity (from left to right). *N* represents the number of molecules in the aggregate.

From here, we extended the investigation to the **3,6-*dtb*-DPBFMe** and **3,6-*dtb*-DPBF(Me)**_**2**_ molecules (Figure 6) and to other solvents [MeCN, MeCN/water (40:60 v/v), MeCN/water (25:75 v/v), and water]. Figure 6 shows normalized histograms of maximum cluster size for simulations containing eight molecules of each solute. The influence of the solvent in each of the three solute molecules is similar, with no substantial differences. In pure acetonitrile solutions (left panel of Figure 6), the solute molecules are mostly separated with the monomer and dimer being the most frequent cluster sizes. Via analysis of the results for both MeCN/water mixtures (25:75, 40:60 v/v), a great relative population is attributed to the octamer, with the population of monomers decreasing drastically in comparison to those of the acetonitrile solutions (see Figures S17 and S18). This is apparent as the monomers are only predominant in the beginning of the simulation. Other cluster sizes also reveal a significant relative population in the mixture simulations, which is a result of the dynamic formation of the aggregate throughout the MD simulation. Finally, in pure water solutions, the solute molecules remain associated, a natural consequence of the low solubility of these nonpolar molecules in water, nonetheless, in the form of smaller aggregates (right panel of Figure 6).

**Figure 7:**
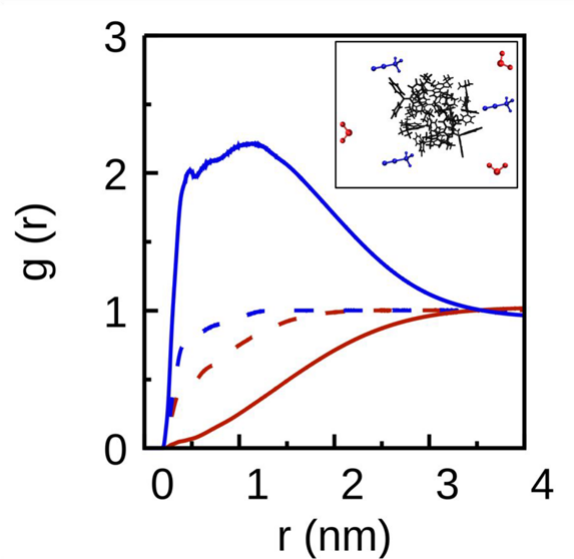
Radial distribution functions of the solvent molecules [pure acetonitrile (dashed blue line), pure water (dashed red line), acetonitrile (solid blue line), and water (solid red line) in a 25:75 MeCN/water mixture] around the **3,6-*dtb*-DPBF** molecules. The inset illustrates the RDF calculated for the **3,6-*dtb*-DPBF** aggregate (black), the water molecules (red), and the acetonitrile molecules (blue).

The distinct results obtained in the different solvents are further explored and rationalized with regard to the solute–solvent intermolecular interactions resorting to the calculation of radial distribution function *g*(*r*). RDF represents the radial probability of finding the corresponding centers of mass of a given molecule at a given distance from a reference molecule and may offer details about solute solvation. As a representative example, the RDFs of the solvent molecules (pure acetonitrile, pure water, and acetonitrile and water in a 25:75 MeCN/water mixture) around the reference solute molecule **3,6-*dtb*-DPBF** (*N* = 8) are represented in Figure 7.

From the water RDF around **3,6-*****dtb*****-DPBF** in the simulation with a MeCN/water mixture (red line from Figure 7), detecting water molecules very close to the solute molecules is unlikely. Actually, the water molecules are preferentially distributed at distances of >3 nm, and below these distances, they are nearly entirely excluded from the solvation shell of the solute. On the contrary, the RDF peak for acetonitrile in the simulation with a MeCN/water mixture (blue line from Figure 7) is located at shorter distances, which may be attributed to stronger solute–solvent interaction at such distances. These observations in the structural organization of both acetonitrile and water molecules in the mixture simulation confirm the influence of the hydrophobic effect in the aggregation of these nonpolar molecules, rationalizing the preferential aggregation in the presence of water. In this way, in the simulations involving MeCN/water mixtures one may deduce that the first solvation shell of the aggregate is mainly constituted of acetonitrile molecules, with the water molecules located preferentially at larger distances. These effects on the solvent distribution around nonpolar solute molecules have been attributed to the hydrophobic effect in previous works.^24,25^

**Figure 8:**
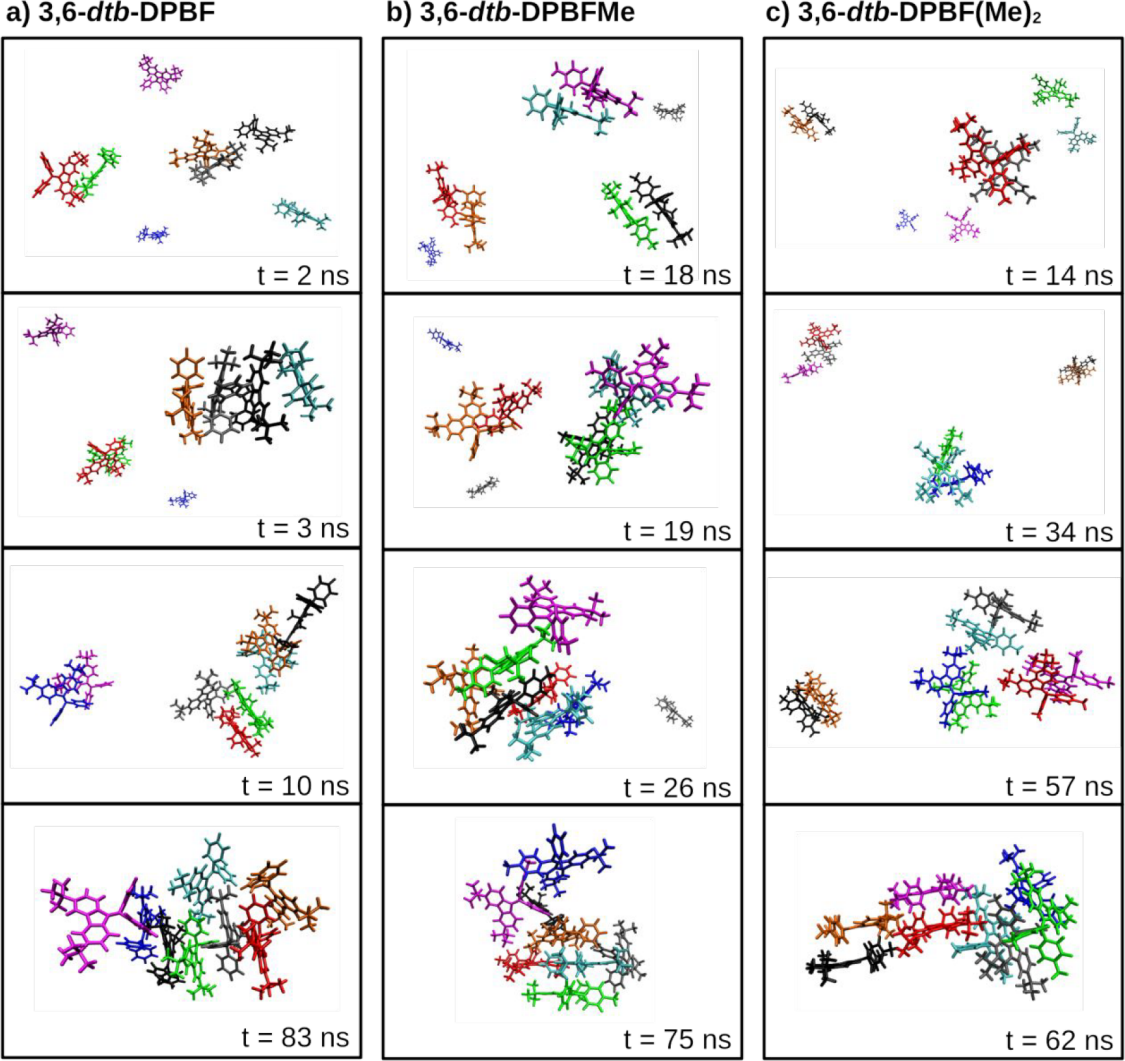
Snapshots of the MD simulation representing the main stages until the aggregation of the eight monomers in the simulations of the (a) **3,6-*dtb*-DPBF**, (b) **3,6-*dtb*-DPBFMe**, and (c) **3,6-*dtb*-DPBFMe**_**2**_ molecules in a 25:75 MeCN/water mixture. Each monomer is represented by a different color. The simulation time is shown in each frame. Snapshots were obtained with the VMD (Visual Molecular Dynamics) program.^26^

From the results mentioned above, the formation of stable **3,6-*dtb*-DPBF**, **3,6-*dtb*-DPBFMe**, and **3,6-*dtb*-DPBF(Me)**_**2**_ aggregates in MeCN/water mixtures is clear. This is confirmed by visual inspection of the MD trajectories in the 25:75 MeCN/water mixture, which is illustrated in Figure 8 with representative snapshots of the dynamical formation of the three aggregates. For complementary information, see Figure S19, in which the cluster/aggregate size throughout the simulation time of the trajectory is represented. The aggregation mechanism is identical for the three selected solute molecules, starting with dimer formation, at the very beginning of the simulation, around 2, 14, and 18 ns. Afterward, the dimers interact with each other or with monomers, rapidly evolving until eight molecules aggregate very early in the simulation, at around 62–83 ns. Furthermore, it is apparent from Figure S18 that the formed clusters remain associated during most of the remaining time of the simulation.

**Figure 9:**
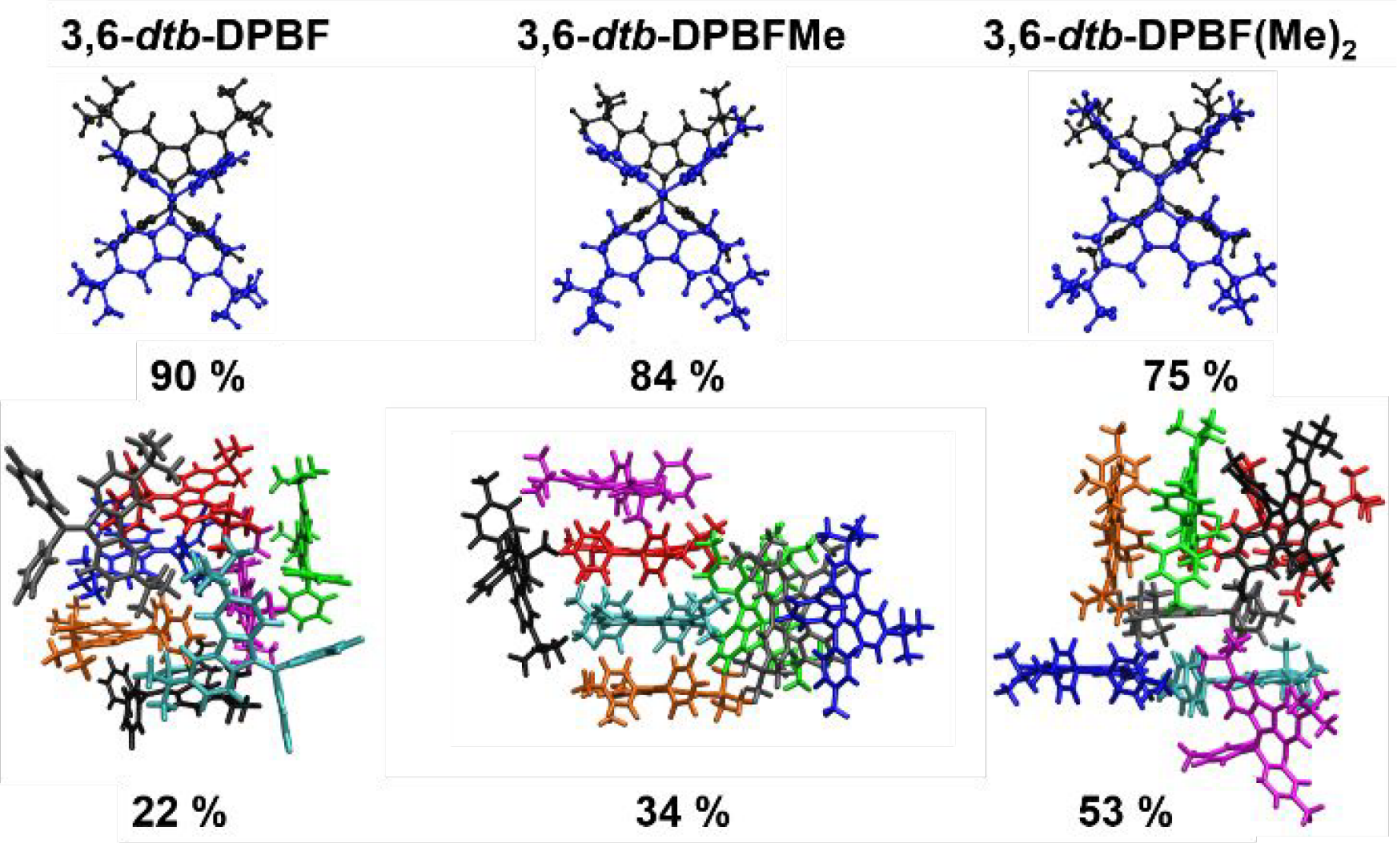
Cluster analysis of two (top) and eight (bottom) monomers in the simulations of the **3,6-*dtb*-DPBF** (left), **3,6-*dtb*-DPBFMe** (middle), and **3,6-*dtb*-DPBF(Me)**_**2**_ (right) molecules in the 25:75 MeCN/water mixture. Each monomer is represented by a different color.

The formed aggregates may assume various shapes due to the dynamic nature of the MD simulations. Nevertheless, in Figure 9, the most probable structures for the **3,6-*dtb*-DPBF**, **3,6-*dtb*-DPBFMe**, and **3,6-*dtb*-DPBF(Me)**_**2**_ dimer and eight-molecule aggregates in the 25:75 MeCN/water mixture simulations are represented. The dimer and octamer structures have been estimated through cluster analysis, considering only the section of the trajectory where the aggregation was already complete. One can notice that the dimer structure is identical for **3,6-*dtb*-DPBF**, **3,6-*dtb*-DPBFMe**, and **3,6-*dtb*-DPBF(Me)**_**2**_ molecules, with the two molecules stacked in an antiparallel motif in relation to the fluorene stator and the phenyl rotors, as already reported for DPBF structures.^7^ The most frequent structures of the octamer are also presented Figure 8. Notice that the insertion of one and two methyl groups appears to increase the cluster probability, from 22% to 34% and 53%, respectively, which may be clear evidence of the increment in the aggregate stability with the introduction of these methyl groups. This stability is also evident in Figure S18, where the cluster size in terms of molecule numbers remains mainly eight for both **3,6-*dtb*-DPBFMe** and **3,6-*dtb*-DPBF(Me)**_**2**_ throughout the entire simulation, whereas the 3,6-*dtb*-DPBF cluster size slightly fluctuates. Moreover, **the 3,6-*dtb*-DPBFMe** also visually appears to display a more structurally organized aggregate, with four molecules in an antiparallel stacking. Indeed, the dimer contribution decreases with the number of methyl groups [from 90% in 3,6-*dtb*-DPBF to 75% in **3,6-*dtb*-DPBF(Me)_2_**] at the expense of the increment in the octamer contribution ([from 22% in 3,6-*dtb*-DPBF to 53% in **3,6-*dtb*-DPBF(Me)_2_**)]. Other structures with lower or insignificant frequency may also be found in the cluster analysis of the MD trajectories. For the octamer structures obtained from the cluster analysis, we calculated aggregate diameters of 2.775, 3.48, and 3.16 nm for **3,6-*dtb*-DPBF**, **3,6-*dtb*-DPBFMe**, and **3,6-*dtb*-DPBF(Me)_2_**, respectively.

### Emission Properties in Thin Films

**Figure 10:**
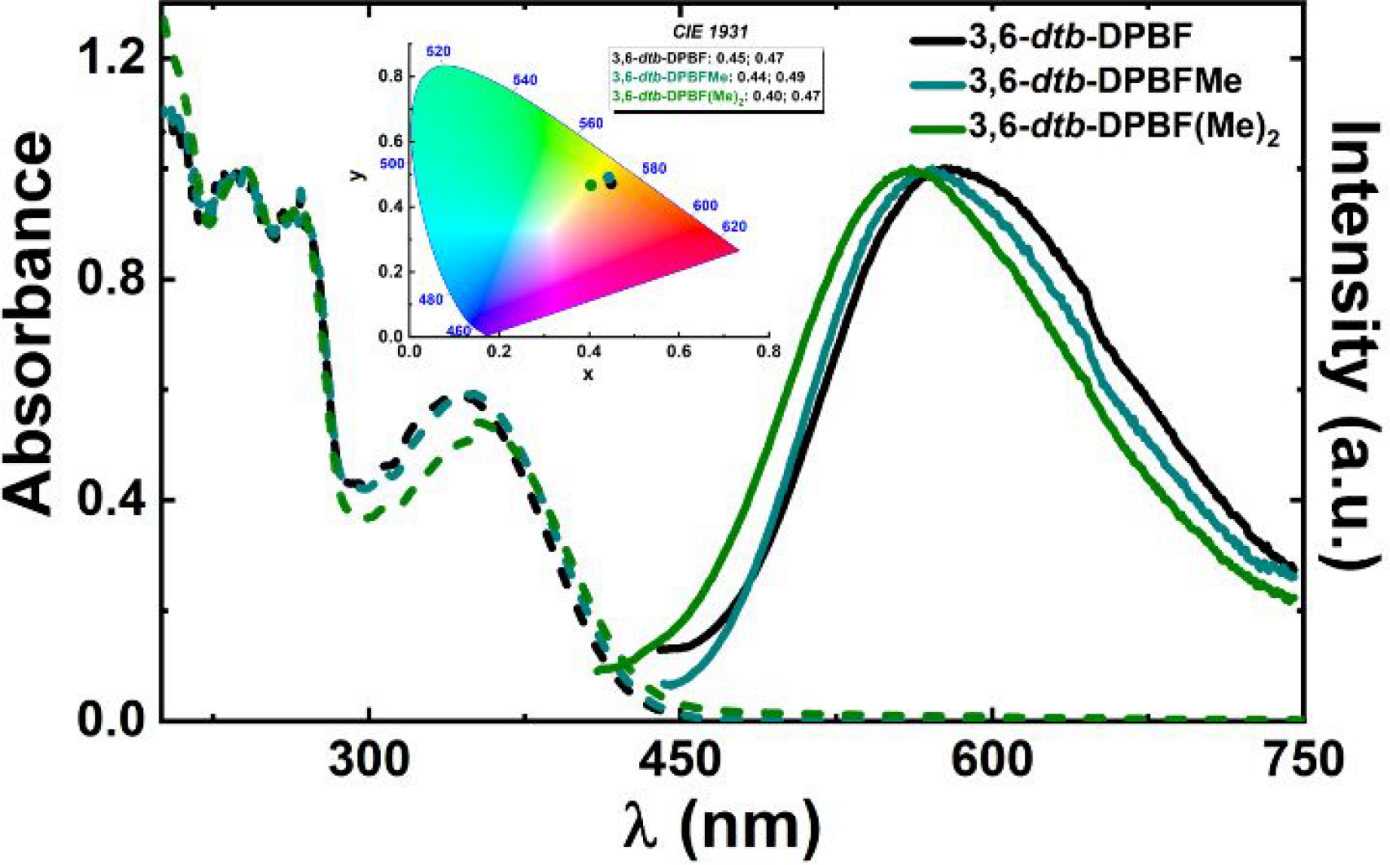
Absorption (dashed line) and emission (solid line) spectra of the 3,6-*dtb*-DPBF derivatives in Zeonex films. Photoluminescence color coordinates of the films are plotted in the CIE 1931 chromaticity diagram.

Thin films constitute an additional and more organized solid-state structure of 3,6-*dtb*-DPBF derivatives. Spin-coated thin films of the compounds were prepared using Zeonex^16^ as a support. The three compounds show similar absorption spectra with a broad band in the range of 300–450 nm, peaking at ∼350 nm (Figure 10 and Table 3), and a broad emission, between 450 and 750 nm. Addition of methyl (**3,6-*dtb*-DPBFMe**) and dimethyl [**3,6-*dtb*-DPBF(Me)**_**2**_] substituents to the *para* position of the biphenyl rotor induces the formation of a broader absorption spectrum, with a blue-shift of the emission spectra. Thin films of the compounds also display large Stokes shifts (Table 3). Moreover, in thin films, **3,6-*dtb*-DPBF(Me)**_**2**_ is more emissive than **3,6-*dtb*-DPBF** and **3,6-*dtb*-DPBFMe** (see Table 3).

**Table 3:** Room-Temperature Spectroscopic Data (absorption and fluorescence emission maxima together with Stokes shifts, Δ_SS_) Along with Fluorescence Quantum Yields (ϕ_F_) for the 3,6-*dtb*-DPBF Derivatives in Thin Films

compound	λ_abs_ (nm)	λ_em_ (nm)	Δ_SS_ (cm^–1^)	ϕ_F_
**DPBF**^a^	341	483	8622	0.044
**3,6-*dtb*-DPBF**	235	577	11739	0.008
	259			
	344			
**3,6-*dtb*-DPBFMe**	236	571	11058	0.011
	263			
	350			
**3,6-*dtb*-DPBF(Me)**_**2**_	241	561	10746	0.013
	266			
	350			

a Data from ref (3).

From the obtained steady-state and time-resolved data, it is possible to observe fluorescence quantum yields much lower than those obtained in solution (with *f*_w_ > 60%) and to observe that the lifetimes are similar to that displayed by the monomer in solution. This shows that different types of aggregates are found in thin films and in solution (with *f*_w_ > 60%), which may be due to a rotational hindering of the monomer and, consequently, nonformation of aggregates. This means that the emissive species is the same with a short associated lifetime (1–2 ns), but when it is fixed on the film, the fluorescence quantum yield decreases dramatically (see Table 3).

It is also interesting to observe that the spectral changes in Figure 2 show that the longest wavelength absorption band of the aggregate species spectra red-shifts and gains vibronic resolution in comparison with that of the monomer, isolated species, in a good solvent (MeCN). Moreover, as mentioned and shown in Figure 10, the absorption spectra in thin films strongly resemble the spectra in the good solvent acetonitrile and are associated with the absorption and emission of monomer species. This spectral feature, i.e., the preferential formation of small order aggregates, is confirmed by the discussed MD data and by the predicted spectra, from TDDFT, of the monomer and dimer (smaller aggregate can be formed). Figure S20 shows the predicted absorption spectra (with oscillator strength) for the optimized monomer and dimer structures of 3,6-*dtb*-DPBF from TDDFT calculations. The dimer was optimized from the structure obtained by molecular dynamics calculations (see Figure 9). Moreover, the spectral maxima of the dimer, predicted from TDDFT calculations, are also blue-shifted compared to those of the monomer.

### Fluorescence Lifetime Imaging Microscopy (FLIM) Studies

**Figure 11:**
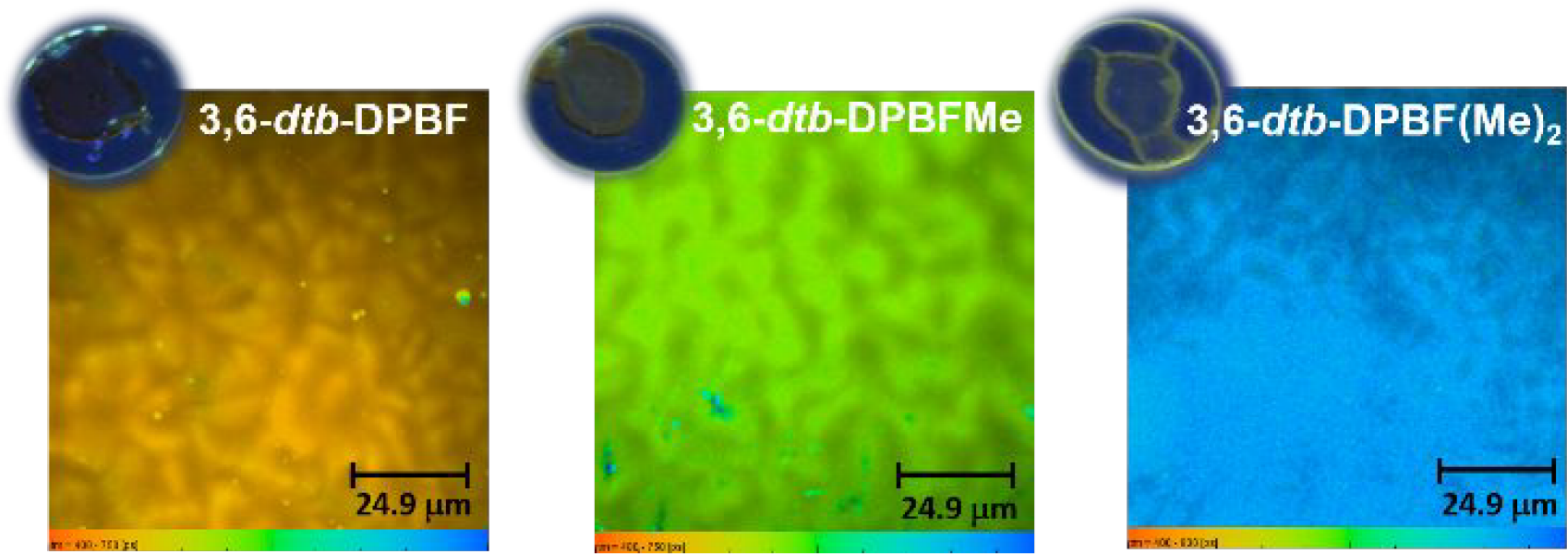
Fluorescence lifetime imaging microscopy (FLIM) images of the 3,6-*dtb*-DPBF derivatives and photographs of the films under ultraviolet excitation (λ_exc_ = 375 nm).

Fluorescence lifetime imaging microscopy (FLIM) studies were performed, and images of the three 3,6-*dtb*-DPBF forms in thin films were obtained and are shown in Figure 11. The data are summarized in Table S8 (average lifetime values). Fluorescence lifetime images (Figure 11) were obtained by analyzing the fluorescence decay curves (following a Gaussian-like distribution) of each pixel, in different frames, using a double-exponential decay function (τ_1_ = 0.34–0.48 ns, and τ_2_ = 1.10–1.68 ns), with similar contributions, and then displaying the FLIM image using a pseudocolor scale (Figure 11, bottom scale). Regardless of the frame (different locations of the film) considered in the acquisition of the fluorescence decays, the average times obtained were similar, which is an indication of the uniformity of the film.

## Conclusion

A study involving 3,6-*dtb*-diphenylbenzofulvene derivatives (newly synthesized) and performed in solution and in the solid state (thin films) shows that the addition of methyl groups to the *para* position of the benzophenone (mono- and disubstituted) moiety leads to an enhancement of fluorescence emission, here shown to be an aggregation-induced emission (AIE) behavior, in acetonitrile/water mixtures. The increase in the fluorescence emission in thin films and in MeCN/water mixtures corroborates the presence of AIE due to the DPBF core. With an increase in the water fraction to >70% H_2_O, a unimodal distribution peaking between 63 and 408 nm, reflecting the formation of aggregates, is observed, which is further linked to the longer decay component obtained in the time-resolved fluorescence data. In agreement with the experimental measurements, the MD results irrefutably indicate the formation of stable 3,6-*dtb*-DPBF derivative aggregates in MeCN/water mixtures and a total separation in acetonitrile solutions. The total number of solute molecules employed in the simulation contributed to the formation of one large-diameter aggregate, which is in agreement with the experimental measures.

## Supporting Information Available

Crystallographic data (CIF)
Materials and instrumentation; experimental procedures; ^1^H NMR, ^13^C NMR, HRMS(ESI), and DLS spectra of the compounds; and steady-state and time-resolved fluorescence data collected as a function of water fraction and in films (PDF)

## Supplementary Material

ot2c00067_si_001.cif

## Abbreviations

DPBF: diphenyldibenzofulvene
*f*_w_: water fraction
MeCN: acetonitrile
