# Supplementary material for: Practical Design of 3,6-Di-tert-butyldiphenyldibenzofulvene Derivatives with Enhanced Aggregation-Induced Emission
Source: ACS Appl Opt Mater. 2022 Nov 10;1(1):340–53. doi: 10.1021/acsaom.2c00067 (PMC9903363; doi:10.1021/acsaom.2c00067)
Supplement: Supplementary file 2 — ot2c00067_si_002.pdf [file ot2c00067_si_002.pdf]

Supporting Information

Practical Design of 3,6-di-*tert*-butyl-diphenyldibenzofulvene Derivatives with Enhanced Aggregation-Induced Emission

*Carla Cunha+, Mariana S. Peixoto+, Joana Santos+, Paulo E. Abreu+, José A. Paixão†, Marta Pineiro+ and J. Sérgio Seixas de Melo+**

[a]*University of Coimbra, CQC, Department of Chemistry, Rua Larga, Coimbra 3004-535, Portugal.*

[b]*University of Coimbra, CFisUC, Department of Physics, Rua Larga, Coimbra 3004-516, Portugal.*

*email: sseixas@ci.uc.pt (J. Sérgio Seixas de Melo)

Keywords: 3,6-di-*tert*-butylfluorene, DPBF, aggregation-induced emission, time-resolved fluorescence, DLS, FLIM, molecular dynamics (MD) simulations

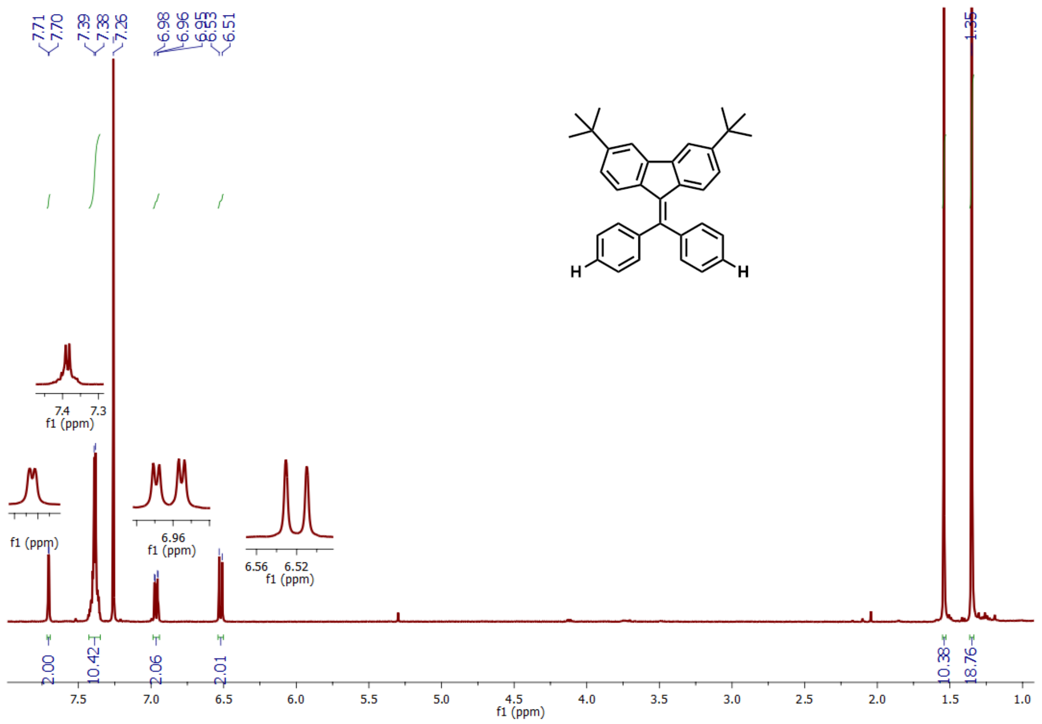

Figure S1. ^{1}H NMR spectrum of **3,6-*dtb*-DPBF** in $CDCl_3$.

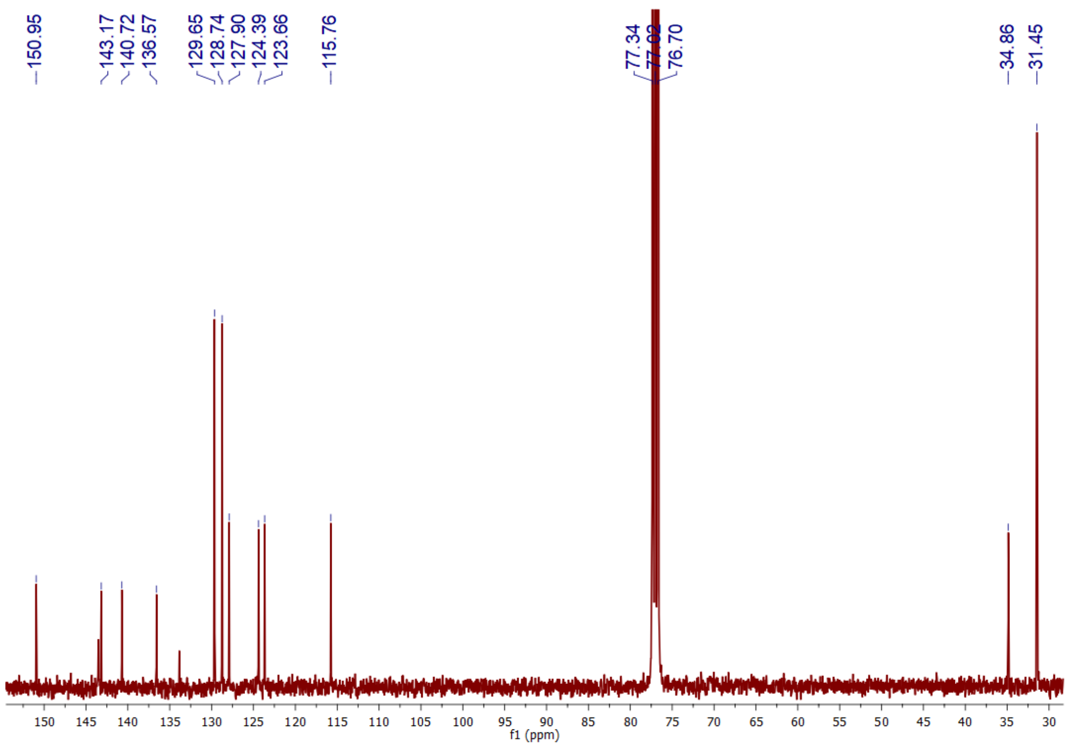

Figure S2. ^{13}C NMR spectrum of **3,6-*dtb*-DPBF** in $CDCl_3$.

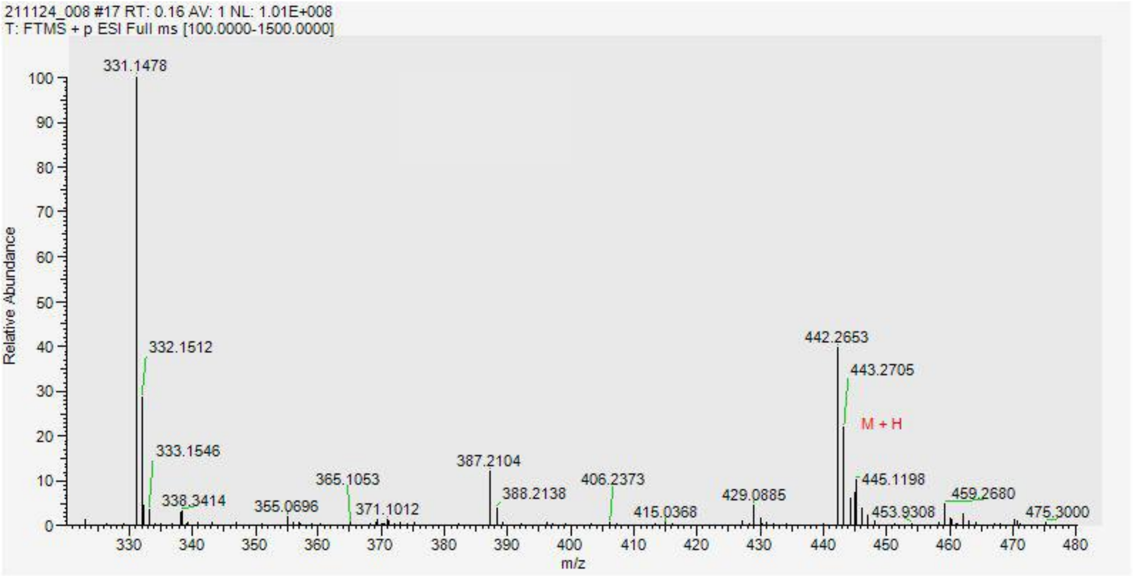

Figure S3. High-resolution mass spectrum of **3,6-*dtb*-DPBF.**

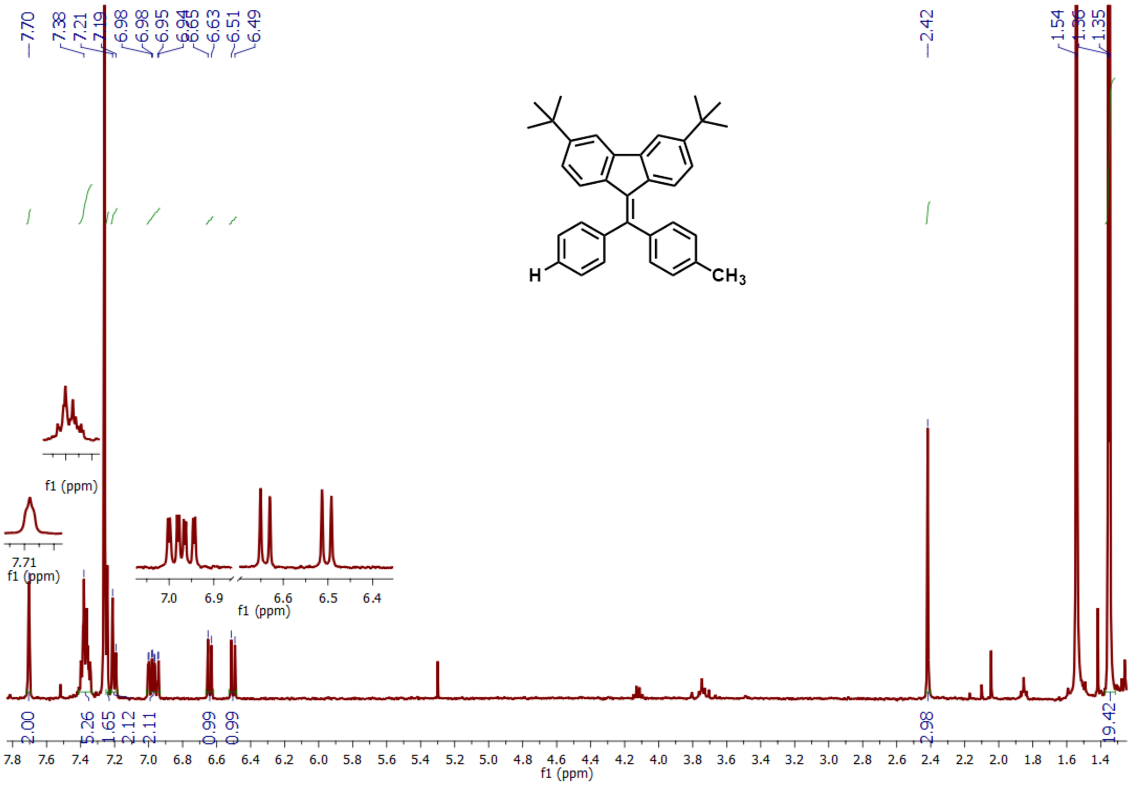

Figure S4. ^{1}H NMR spectrum of **3,6-*dtb*-DPBFMe** in $CDCl_3$.

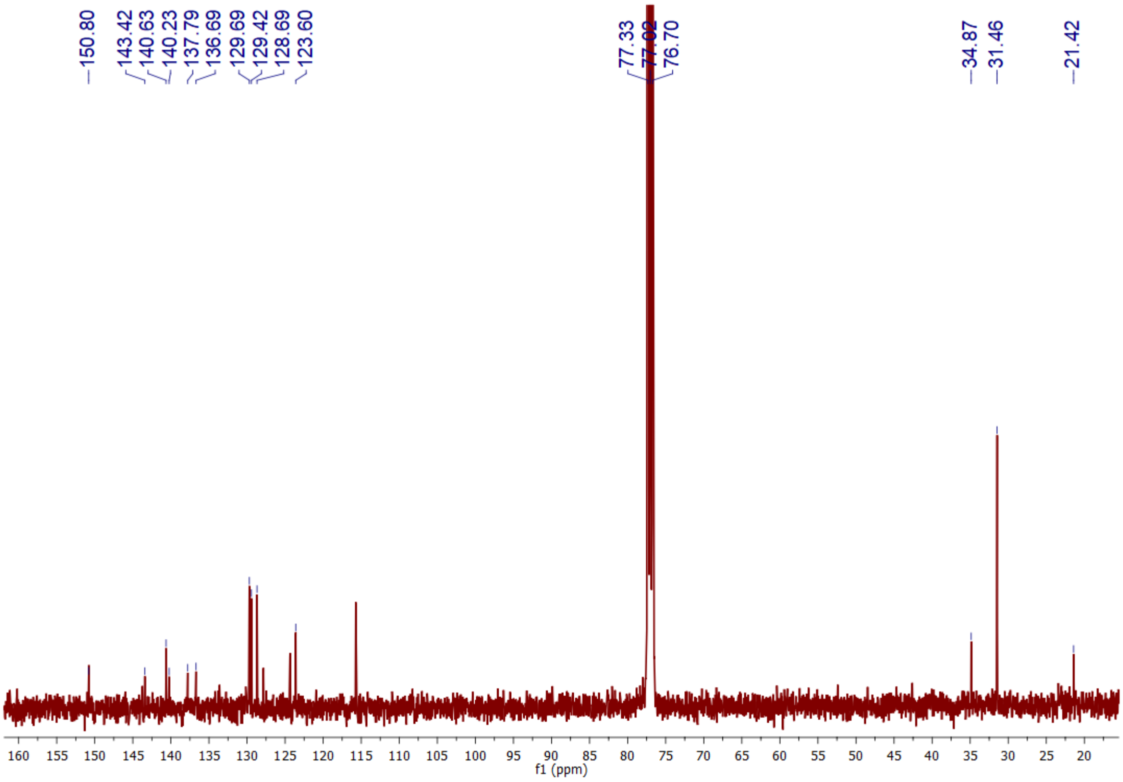

Figure S5. ^{13}C NMR spectrum of **3,6-*dtb*-DPBFMe** in $CDCl_3$.

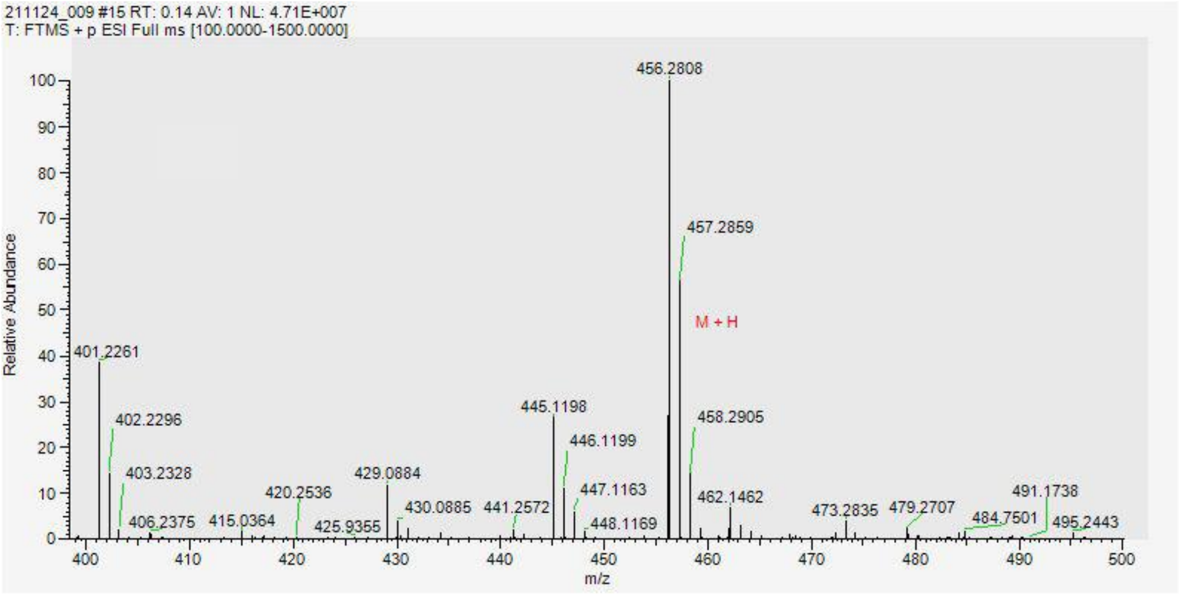

Figure S6. High-resolution mass spectrum of **3,6-*dtb*-DPBFMe.**

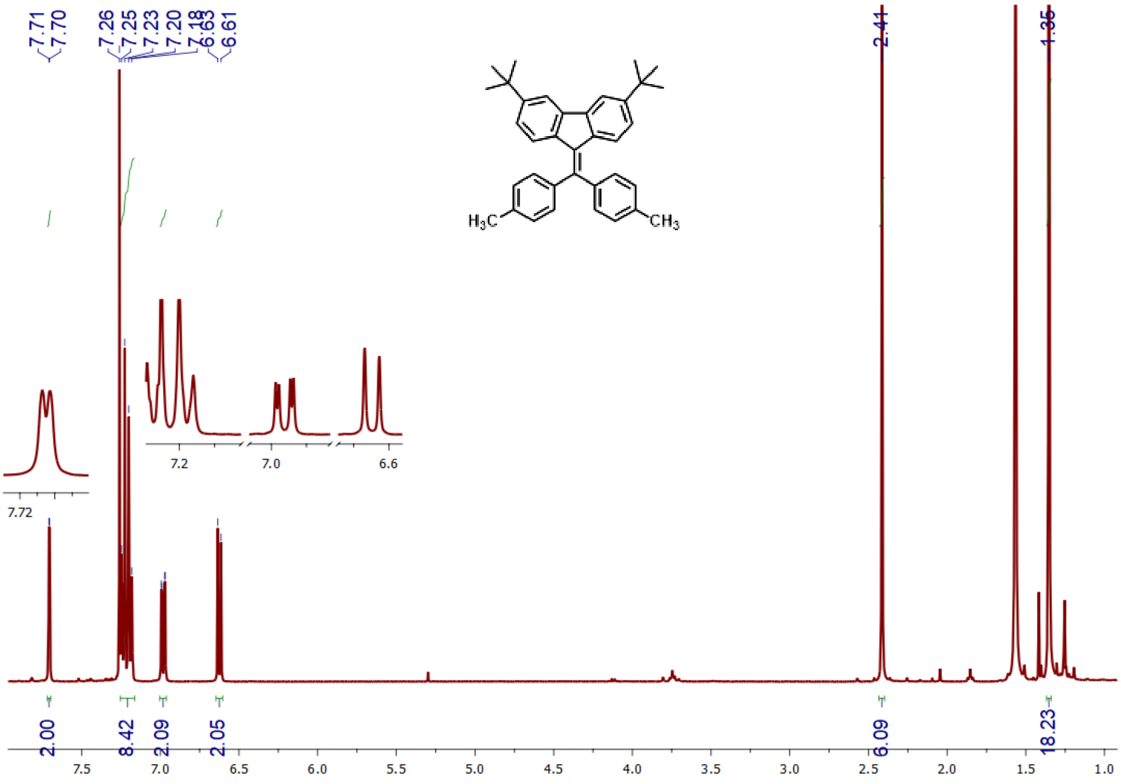

Figure S7. ^{1}H NMR spectrum of ***3,6-dtb*-DPBF(Me)$_2$** in $CDCl_3$.

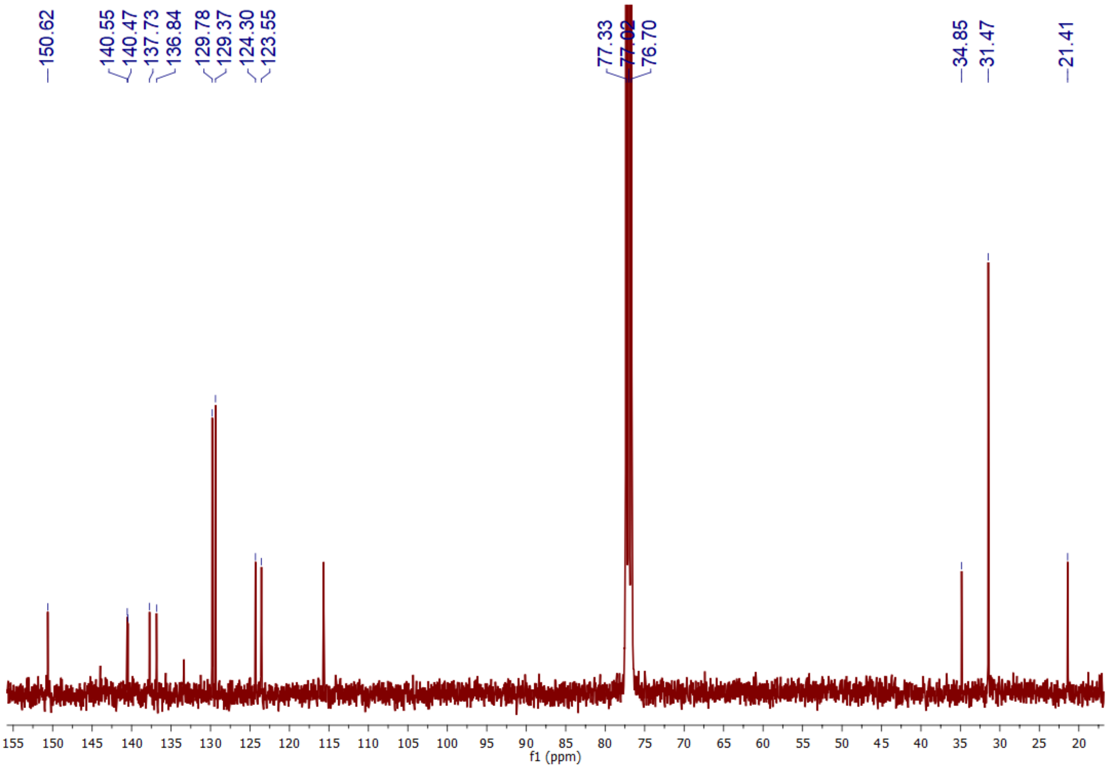

Figure S8. ^{13}C NMR spectrum of ***3,6-dtb*-DPBF(Me)$_2$** in $CDCl_3$.

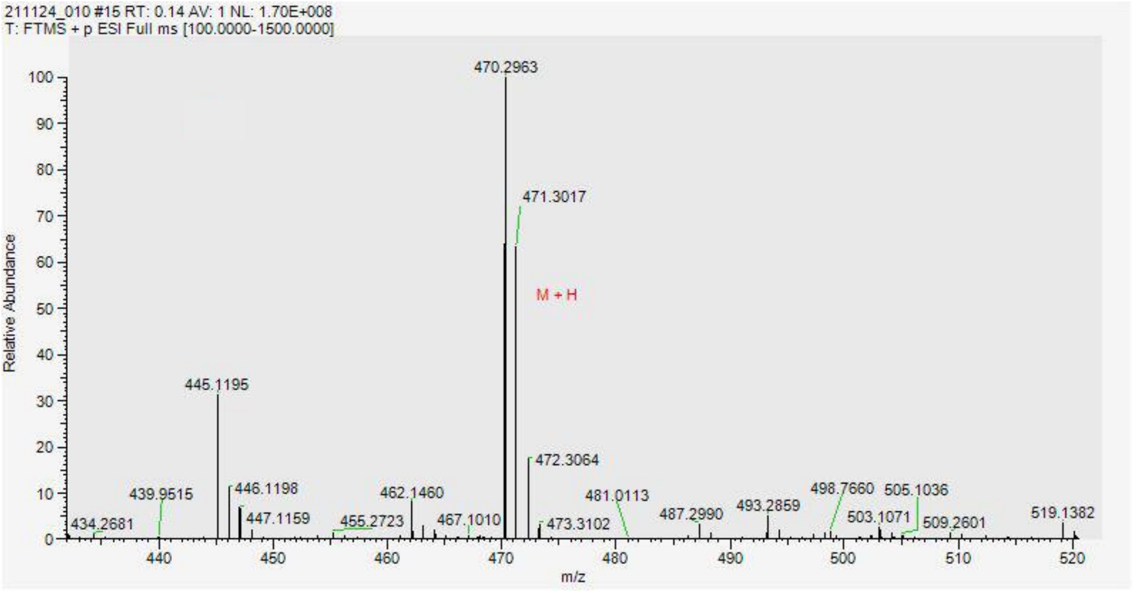

Figure S9. High resolution mass spectrum of **3,6-*dtb*-DPBF(Me)$_2$.**

Table S1. Summary of the single-crystal X-ray data collection and crystal structure refinement.

Chemical formula	$C_{34}H_{34}$
Formula weight	442.61
Color, shape	Light yellow/needle
Space group	*P*-1
Temperature(K)	293(2)
Cell volume (Å^3)	1322.6(4)
Crystal system	triclinic
a (Å)	9.8280(14)
b (Å)	11.8179(17)
c (Å)	12.084(2)
α (deg)	84.514(11)
β (deg)	71.237(11)
γ (deg)	89.341(11)
Z,Z´	2,1
D_c (Mg m^{-3})	1.111
Radiation (Å) (graphite; monochromated)	0.71073
Max. crystal dimensions (mm)	0.22×0.08×0.08
θ range (deg)	2.833 – 24.999
Range of *h*, *k*, *l*	–11,11;–14,14;–13,14
Reflections measured/independent	29500/4642
Reflections observed ($I > 2\sigma$)	2315
Data/restraints/parameters	4642/0/314
GOF	1.059
R_1 ($I > 2\sigma$)	0.1006
wR_2	02694
Function minimized	$\Sigma w (\|F_o\|^2 - S\|F_c\|^2)$
Diff. density final max/min (e Å^{-3})	0.351, –0.366

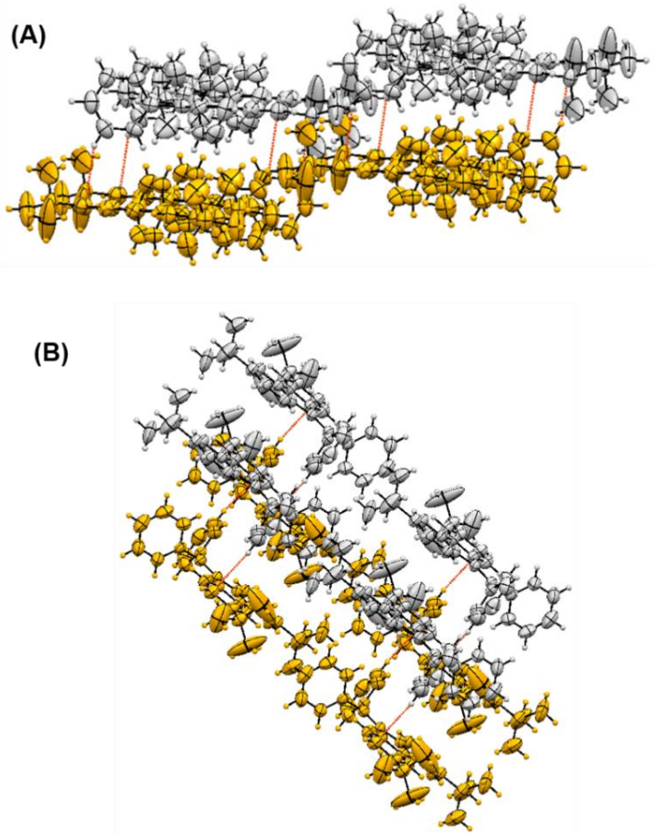

Figure S10. (**A**) Top view and (**B**) front view of the structure of **3,6-*dtb*-DPBF** with relevant intermolecular short contacts highlighted in red (C···H= 2.857 Å).

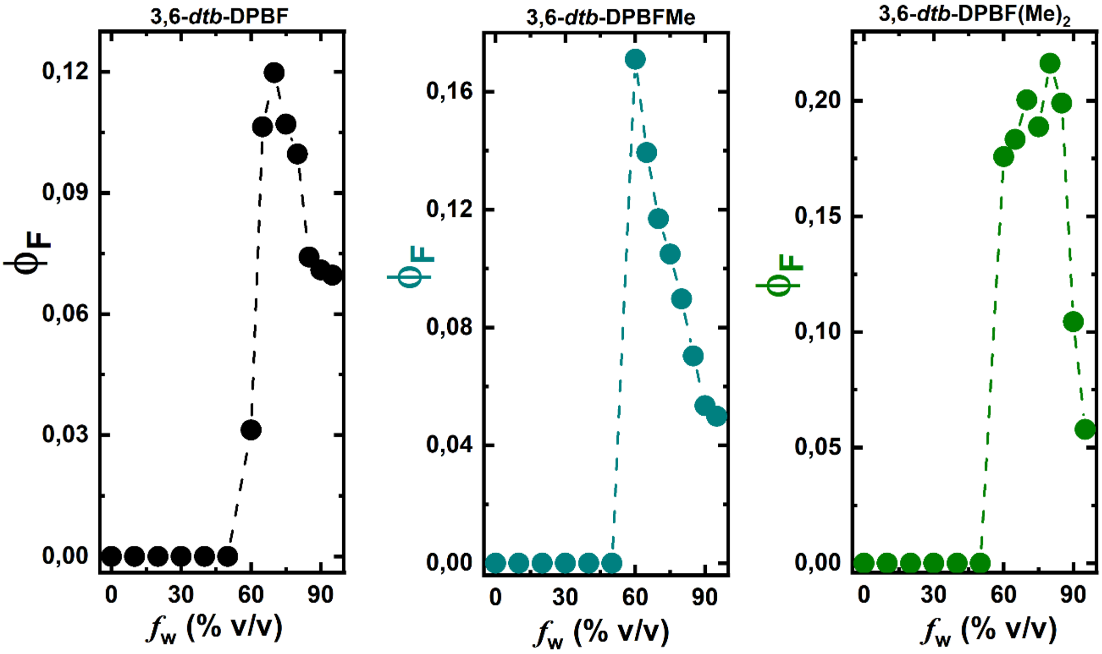

Figure S11. Fluorescence quantum yield with increasing water fraction, f_W = 0–95%.

Table S2. Photoluminescence emission color coordinates of 3,6-*dtb*-diphenyldibenzofulvenes derivatives in acetonitrile/water mixtures, with different water fractions.

Compound	f_w (% v/v)	x	y
**3,6-*dtb*-DPBF**	0	0.43	0.37
	75	0.19	0.24
**3,6-*dtb*-DPBFMe**	0	0.24	0.18
	60	0.19	0.25
**3,6-*dtb*-DPBF(Me)$_2$**	0	0.34	0.31
	80	0.18	0.22

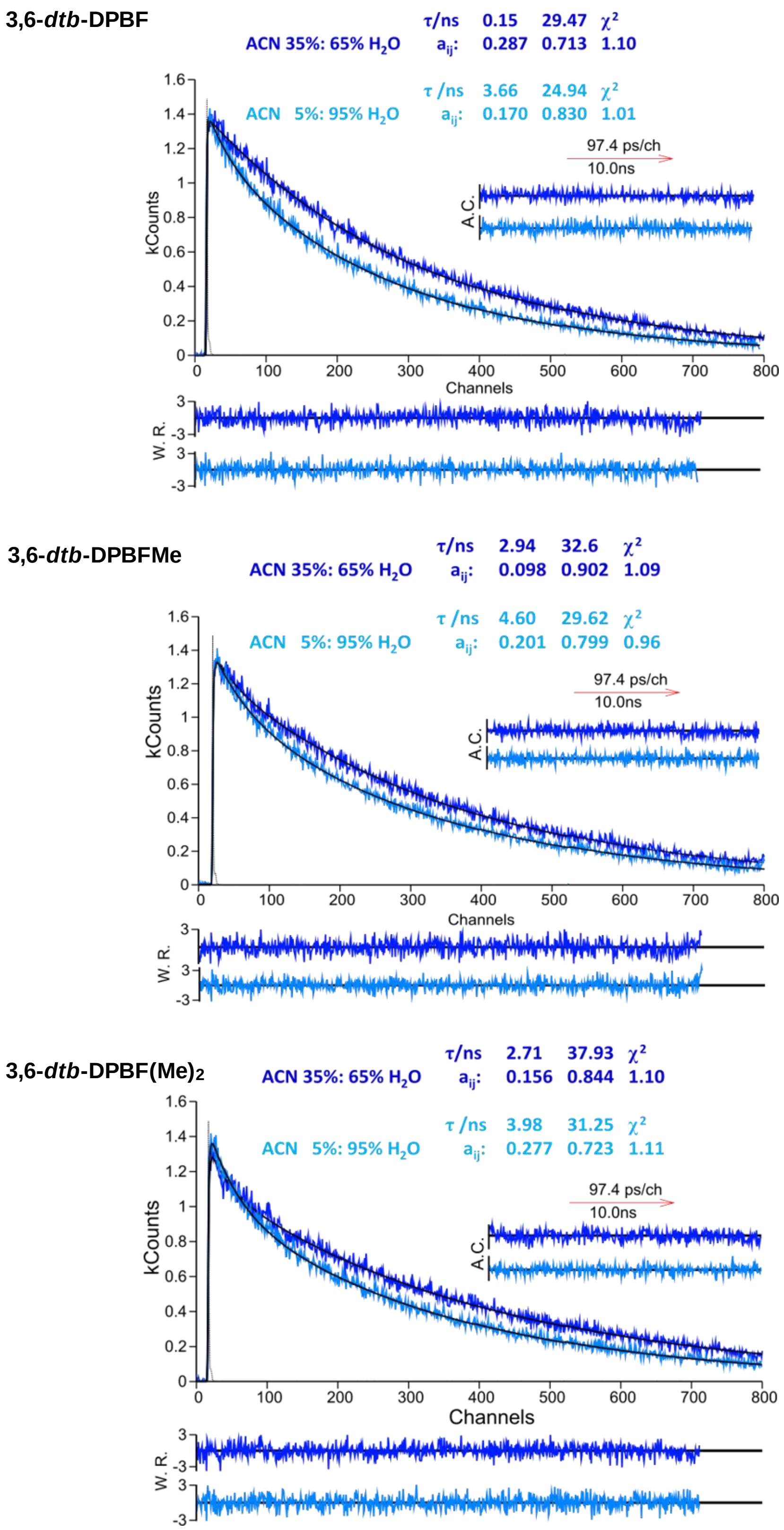

Figure S12. Fluorescence decays for the **3,6-*dtb*-DPBF** derivatives in MeCN/water mixtures, with different water fractions, at T=293 K, with λ_{exc} = 261 nm and λ_{em} = 470 nm. For a better judgment of the quality of the fit, weighted residuals (W.R.), autocorrelation function (A.C.) and χ^2 values are also presented. The dashed line in the decay of corresponds to the instrumental response function.

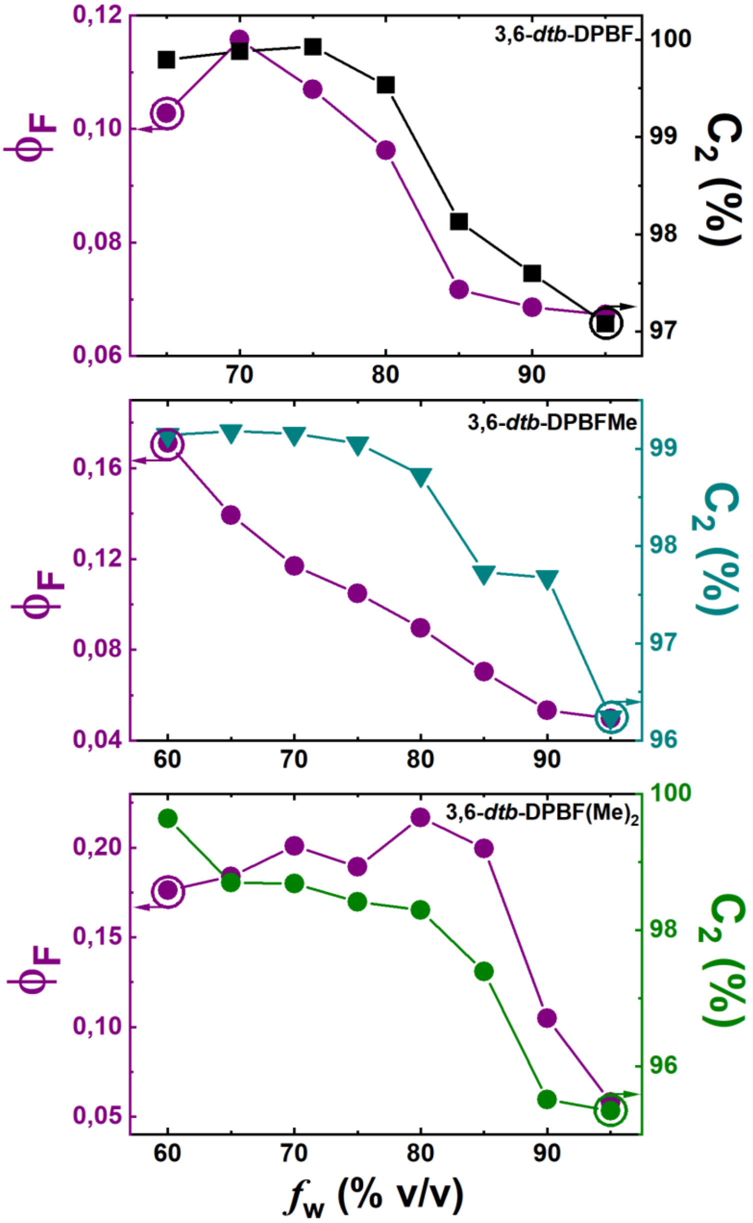

Figure S13. τ_2 contributions (C_2 in %) and PL quantum yields (ϕ_F) for 3,6-*dtb*-DPBF derivatives in acetonitrile/water mixtures with increasing water fraction, f_w. The plot aims at demonstrating the correlation between these two parameters, see the text for more details.

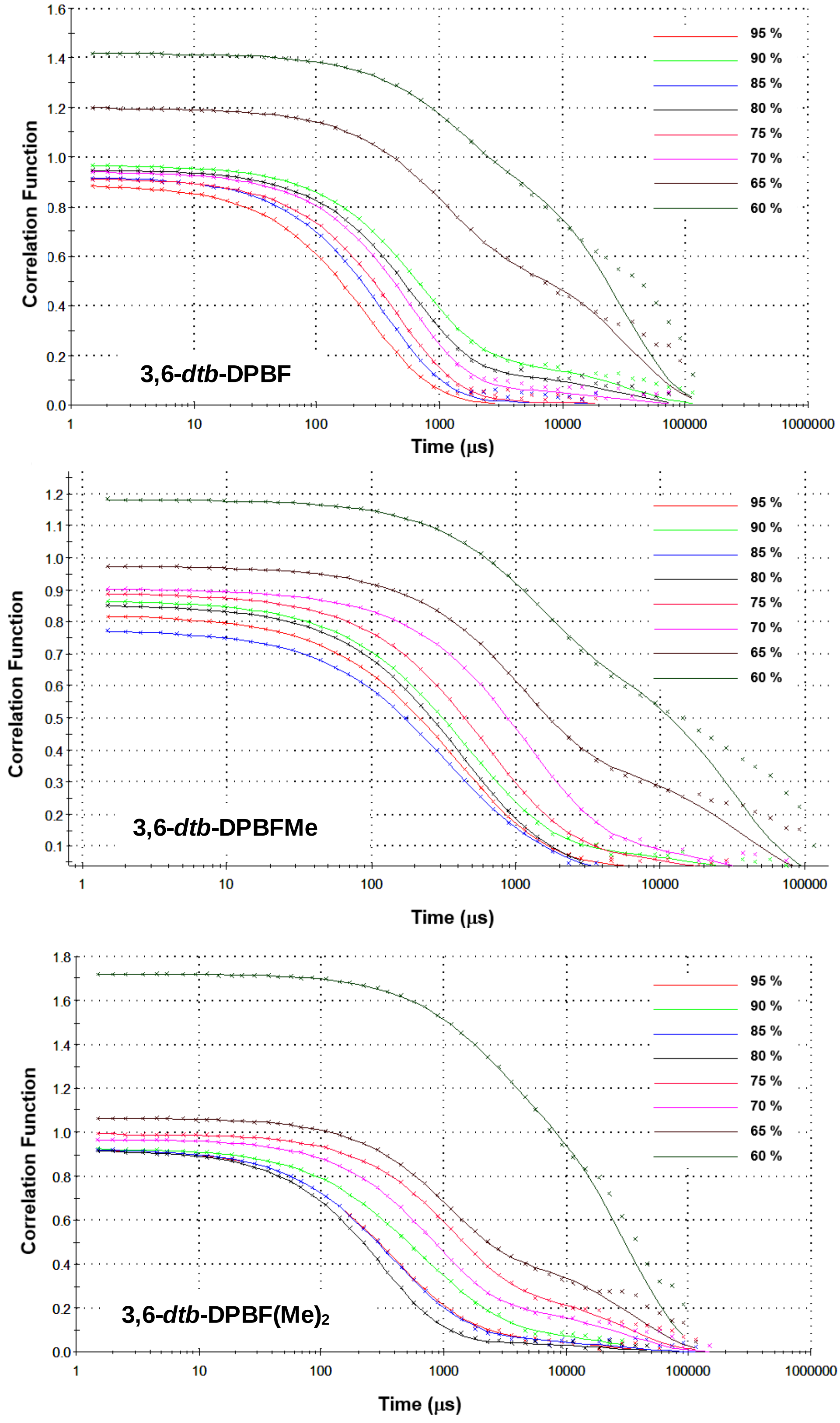

Figure S14. Autocorrelation functions for 3,6-*dtb*-DPBF derivatives in the acetonitrile/water mixture (% v/v), with different water fractions.

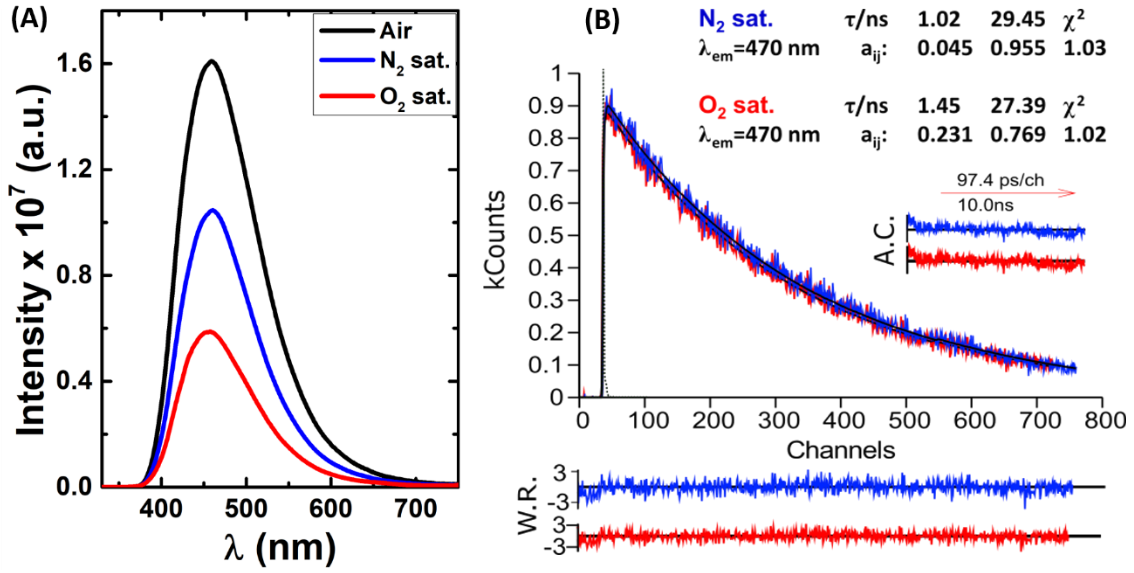

Figure S15. (**A**) Fluorescence emission spectra with air (black line), N_2 saturated (blue line) and O_2 saturated (red line) and (**B**) Time resolved fluorescence of the PL decay times (τ_i), an example **3,6-*dtb*-DPBF**, obtained with ps-TCSPC technique in solutions with N_2 saturated (blue line) and O_2 saturated (red line); λ_{exc} = 261 nm and λ_{em} = 470 nm at T = 293 K. For a better judgment of the quality of the fit, weighted residuals (W.R.), autocorrelation function (A.C.) and χ^2 values are also presented. The dashed line in the decay of corresponds to the instrumental response function.

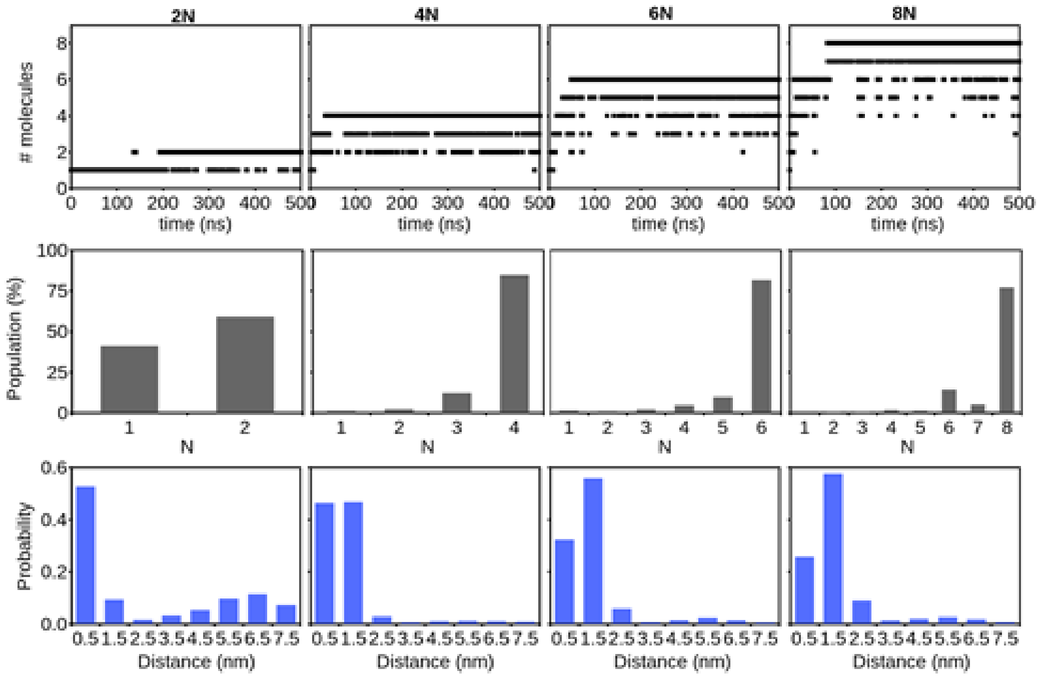

Figure S16. (Upper) Evolution of the maximum cluster size of the **3,6-*dtb*-DPBF** systems during the MD simulations in the 25: 75 MeCN/water mixtures. Note that each time step corresponds to one point; (Middle) Histograms of cluster size showing the relative population of molecules in aggregates with different size, with N representing the number of molecules in the aggregate.; (Bellow) Average distances between the various molecules of the system during the simulation.

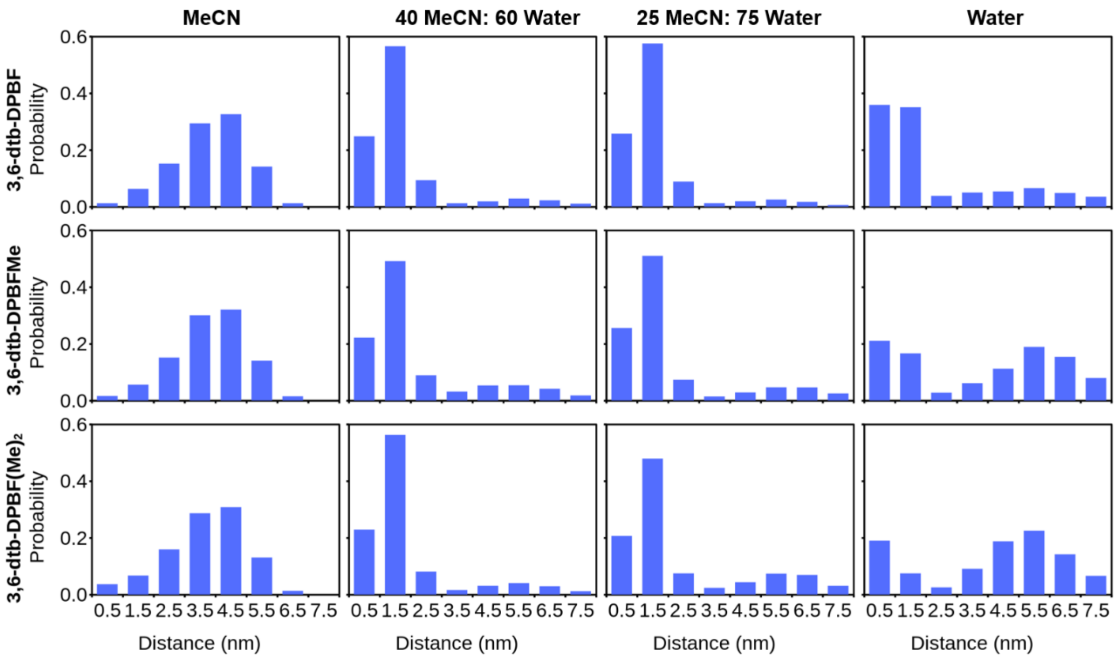

Figure S17. Average distances between the various molecules of the systems (solutes: **3,6-*dtb*-DPBF**; **3,6-*dtb*-DPBFMe**; **3,6-*dtb*-DPBF(Me)$_2$**) in pure acetonitrile, 40:60 and 25:75 MeCN/water mixtures and pure water, during the simulation. The plots are in order of increasing polarity (from left to right).

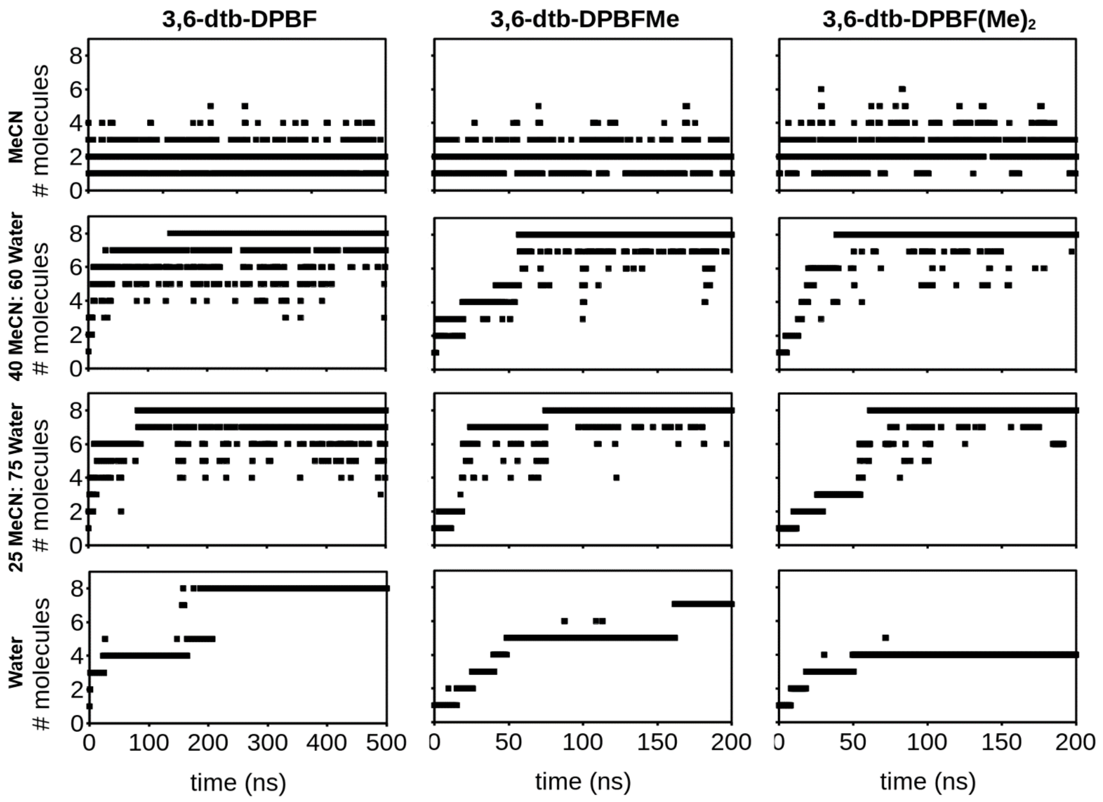

Figure S18. Evolution of the maximum cluster size of the studied systems (solutes: **3,6-*dtb*-DPBF**, **3,6-*dtb*-DPBFMe** and **3,6-*dtb*-DPBF(Me)$_2$**) in pure acetonitrile, 40:60 and 25:75 MeCN/water mixtures and pure water, during the MD simulations. The plots are in order of increasing polarity (from top to bottom)

Table S3. Composition, box side and concentration (c) of each system employed in the MD simulations, at 298.15 K.

Solute	# molecules	c (M)	Simulation time (ns)	Box side (nm)
3,6-*dtb*-DPBF	2	0.0025	500	10.99300
	4	0.0050		
	6	0.0075		
	8	0.0100		
3,6-*dtb*-DPBFMe	8	0.0100	200	
3,6-*dtb*-DPBF(Me)$_2$	8	0.0100	200	

Table S4. Solvent composition employed in each system from **Table S3**.

Acetonitrile (mL v/v)	Water (mL v/v)
0	100
25	75
40	60
100	0

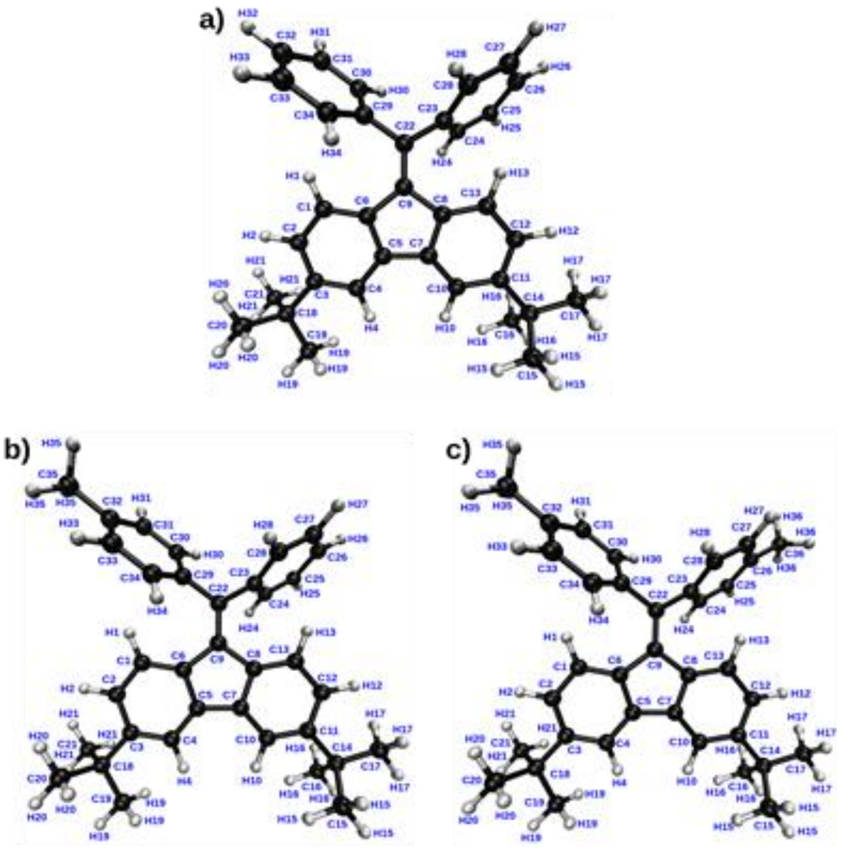

Figure S19. Molecular structures of the solutes molecules used in molecular dynamics simulations. a) **3,6-*dtb*-DPBF**; b) **3,6-*dtb*-DPBFMe** and c) **3,6-*dtb*-DPBF(Me)$_2$**.

Table S5. Partial atomic charges used in the simulations for the **3,6-*dtb*-DPBF** molecule.

**3,6-*dtb*-DPBF**							
**Atom**	**Charge**	**Atom**	**Charge**	**Atom**	**Charge**	**Atom**	**Charge**
C1	-0.193500	C15	-0.307100	C29	0.013400	H17	0.049100
C2	-0.256700	C16	-0.322900	C30	-0.077700	H19	0.057100
C3	0.000100	C17	-0.222300	C31	-0.219100	H20	0.066700
C4	-0.192000	C18	0.393300	C32	-0.094900	H21	0.070900
C5	0.003000	C19	-0.257600	C33	-0.209600	H24	0.125500
C6	0.042200	C20	-0.302900	C34	-0.062700	H25	0.145300
C7	0.026500	C21	-0.318000	H1	0.175100	H26	0.137600
C8	0.053900	C22	-0.061600	H2	0.175700	H27	0.151400
C9	-0.000900	C23	-0.004300	H4	0.133000	H28	0.136900
C10	-0.282900	C24	-0.064500	H10	0.198500	H30	0.128900
C11	0.001700	C25	-0.190600	H12	0.138100	H31	0.157200
C12	-0.165500	C26	-0.110200	H13	0.177600	H32	0.136900
C13	-0.237800	C27	-0.197100	H15	0.067400	H33	0.147700
C14	0.374600	C28	-0.093000	H16	0.071600	H34	0.122900

Table S6. Partial atomic charges used in the simulations for the **3,6-*dtb*-DPBFMe** molecule

**3,6-*dtb*-DPBFMe**							
**Atom**	**Charge**	**Atom**	**Charge**	**Atom**	**Charge**	**Atom**	**Charge**
C1	-0.179700	C16	-0.303900	C31	-0.248500	H20	0.074800
C2	-0.256000	C17	-0.234400	C32	0.163100	H21	0.072400
C3	0.024700	C18	0.359900	C33	-0.241200	H24	0.123900
C4	-0.196900	C19	-0.276400	C34	-0.107400	H25	0.147600
C5	0.008200	C20	-0.325200	C35	-0.249400	H26	0.136100
C6	0.041500	C21	-0.315200	H1	0.156400	H27	0.152100
C7	0.042800	C22	-0.037400	H2	0.174800	H28	0.134400
C8	0.038900	C23	0.004200	H4	0.129300	H30	0.139300
C9	-0.019700	C24	-0.073000	H10	0.199000	H31	0.159000
C10	-0.296000	C25	-0.192900	H12	0.134700	H33	0.150500
C11	0.018000	C26	-0.106100	H13	0.171100	H34	0.130300
C12	-0.174700	C27	-0.196100	H15	0.074800	H35	0.079700
C13	-0.217400	C28	-0.101500	H16	0.067400		
C14	0.366000	C29	0.040700	H17	0.053400		
C15	-0.333800	C30	-0.125000	H19	0.064600		

Table S7. Partial atomic charges used in the simulations for the **3,6-*dtb*-DPBF(Me)2** molecule

3,6-*dtb*-DPBF(Me)2							
**Atom**	**Charge**	**Atom**	**Charge**	**Atom**	**Charge**	**Atom**	**Charge**
C1	-0.158600	C16	-0.306600	C31	-0.230100	H19	0.060100
C2	-0.273000	C17	-0.210800	C32	0.162900	H20	0.068900
C3	0.005400	C18	0.387800	C33	-0.257300	H21	0.067400
C4	-0.196800	C19	-0.267800	C34	-0.093000	H24	0.143800
C5	0.018700	C20	-0.309100	C35	-0.249300	H25	0.156800
C6	0.021300	C21	-0.302700	C36	-0.220400	H27	0.148300
C7	0.043300	C22	-0.025800	H1	0.158100	H28	0.148800
C8	0.027300	C23	0.002800	H2	0.176800	H30	0.148200
C9	-0.023300	C24	-0.112800	H4	0.128900	H31	0.153500
C10	-0.298500	C25	-0.244700	H10	0.201800	H33	0.153800
C11	0.004200	C26	0.142700	H12	0.144700	H34	0.132500
C12	-0.201200	C27	-0.214600	H13	0.159400	H35	0.079900
C13	-0.181000	C28	-0.139000	H15	0.061400	H36	0.073000
C14	0.386600	C29	0.028300	H16	0.065700		
C15	-0.290500	C30	-0.142800	H17	0.044600		

Table S8. Fluorescence decay components for the 3,6-*dtb*-DPBF derivatives in the solid state (thin films), obtained at T = 293 K with λ_{exc} = 375 nm.

**Compound**	**λ_{exc} (nm)**	**τ_1 (ns)**	**τ_2 (ns)**	**a_1**	**a_2**	**χ^2**
**3,6-*dtb*-DPBF**	375	0.34	1.10	85.53 (61)	16.94 (39)	0.89
**3,6-*dtb*-DPBFMe**	375	0.41	1.31	81.41 (58)	18.59 (42)	1.07
**3,6-*dtb*-DPBF(Me)2**	375	0.48	1.68	80.76 (55)	19.24 (46)	1.06

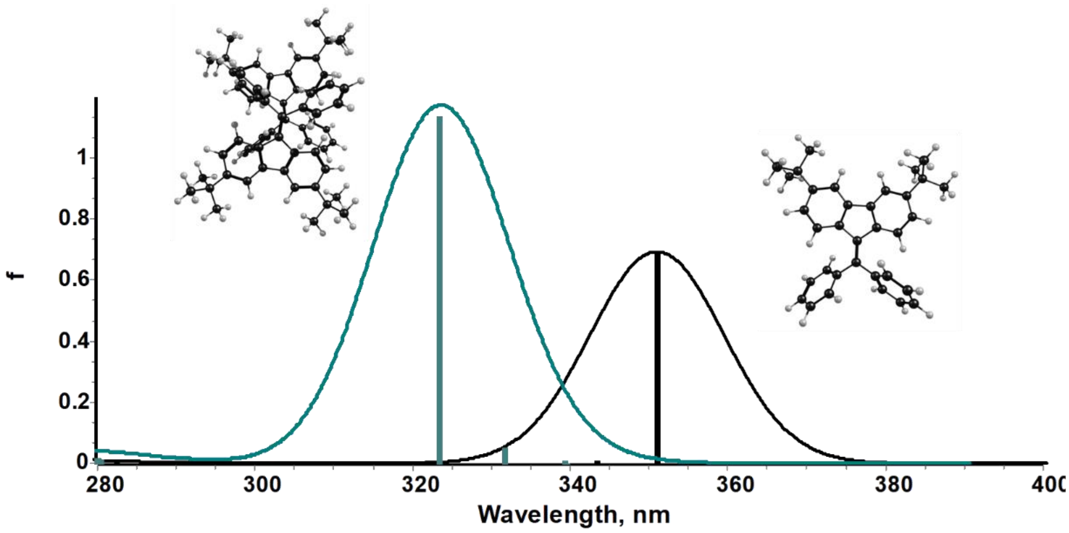

Figure S20. TDDFT absorption spectra of the monomer and dimer species, in acetonitrile, of 3,6-*dtb*-DPBF. Color legend: black – monomer; blue – dimer.
